# [*closo*-B_10_H_8_-10-PhI-1-COOH]^−^ Anion: An Intermediate for Functional Anionic Carboxylate Ligands

**DOI:** 10.1021/acs.inorgchem.4c02044

**Published:** 2024-07-12

**Authors:** Rafał Jakubowski, Szymon Kapuściński, Oleksandr Hietsoi, Andrienne C. Friedli, Piotr Kaszyński

**Affiliations:** † Department of Chemistry, Middle Tennessee State University, Murfreesboro, Tennessee 37132, United States; ‡ Centre of Molecular and Macromolecular Studies, Polish Academy of Sciences, Sienkiewicza 112, 90-363 Łódź, Poland; § Faculty of Chemistry, University of Łódź, Tamka 12, 91-403 Łódź, Poland

## Abstract

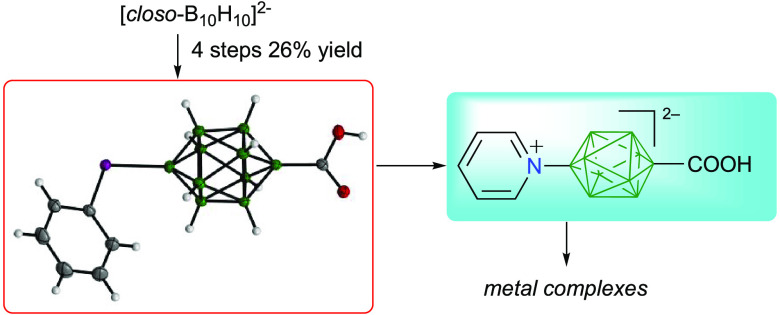

A potentially general intermediate, [*closo*-B_10_H_8_-10-PhI-1-COOH]^–^, for a class of functional anionic carboxylic acids, [*closo*-B_10_H_8_-10-X-1-COOH]^2–^, was obtained in four steps and 26% overall yield from [*closo*-B_10_H_10_]^2–^. It was converted to the pyridinium derivative (X = C_5_H_5_N^+^) and subsequently to coordination complexes with (phen)_2_Cu^2+^ and (phen)_2_Zn^2+^ ions. Both the acid and Zn(II) complex exhibit a cage-to-pyridine charge-transfer band. The availability of such acids opens access to functional metal-ion complexes with compensated charges.

## Abstract 2

The acid [*closo*-B_10_H_8_-10-PhI-1-COOH]^2−^ can be obtained in four steps and 26% overall yield and serves as a synthetic intermediate to functional carboxylic acids, [*closo*-B_10_H_8_-10-X-1-COOH]^2−^, as demonstrated for X = pyridine and its two metal salts.

One of the most versatile functional groups in synthetic chemistry, biology, and materials is the carboxylic acid (COOH) group.^1^ COOH also serves as a bidentate ligand in a variety of metal complexes,^2−7^ including single-molecule magnets,^8−11^ and is one of the key structural elements of metal–organic framework systems.^12−19^ For these reasons, access to new functional carboxylic acids and those with unusual properties remains of continued importance and interest.

Carboxylic acids derived from the [*closo*-B_10_H_10_]^2–^ anion (**A**),^20−22^ such as diacid **1a** (Figure 1),^23,24^ are most unusual: the high negative charge density on the {*closo*-B_10_} cluster makes the carboxyl group weakly acidic^24^ and easy to protonate,^25^ which presumably indicates a facile coordination of metal ions. The latter feature opens up the possibility of accessing metal complexes with functional carboxylic acids **1** possessing an additional cage-delocalized negative charge (a total of 2– or 3– for the carboxylate anion, depending on X). For instance, we have demonstrated intramolecular photoinduced charge-transfer (CT) behavior of pyridinium derivatives of **A**,^26^ which suggests the possibility of photocontrolled basicity of the carboxyl group and metal binding properties if a properly functionalized carboxylic acid is used. Unfortunately, carboxylic acids of the general structure [*closo*-B_10_H_8_-10-X-1-COOH]^2–^ are limited to only three derivatives in addition to the dicarboxylic acid **1a**: 10-cyano **1b**,^24^ 10-(B_10_H_9_-1-CO) **1c**,^27^ and 10-carbonyl **1d**.^25^ Therefore, there is a need to develop access to a larger pool of carboxylic acids of structure **1** with a broader range of substituents X. Such acids are potentially attractive building blocks for functional materials and other derivatives through functional group transformations.

**Figure 1:**
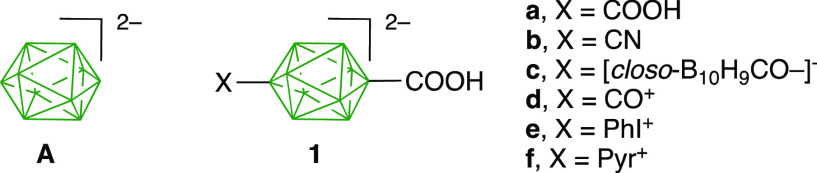
Structures of the parent anion **A** and carboxylic acids **1**. Each unsubstituted green vertex corresponds to a B–H group.

Recently, we described a convenient approach to dicarboxylic acid **1a** through hydrolysis of bis-*N*-methylnitrilium ylide [*closo*-B_10_H_8_-1,10-(CNMe)_2_].^25^ In addition, we have demonstrated an efficient introduction of functional groups, such as CN, AcO, azines, SCHNMe_2_, and I, at the apical position of anion **A** by selective B-phenyliodination, followed by nucleophilic displacement of the PhI group.^26,28−30^

Herein we describe the synthesis of acid [*closo*-B_10_H_8_-10-IPh-1-COOH]^−^ (**1e[Et**_**4**_**N]**) containing the PhI leaving group in the B(10) position as a versatile intermediate for the preparation of B(10)-functionalized carboxylic acids **1**. We demonstrate the utility of **1e[Et**_**4**_**N]** by transformation to 10-pyridinium acid **1f** and the preparation of coordination complexes with Cu(II) and Zn(II). Synthetic work is supported with single-crystal X-ray diffraction (XRD) analysis and augmented with spectroscopic studies.

The preparation of acid **1e[Et**_**4**_**N]** relies on our recently discovered method for activation of the nitrile group toward hydrolysis by N-methylation.^25^ It was determined that the nitrile^26^**2[Et**_**4**_**N]** readily reacts with CF_3_SO_3_Me in CH_2_Cl_2_, giving the nitrilium zwitterion **3** in 68% yield after recrystallization. Interestingly, an attempt to obtain the parent carboxylic acid (X = H) by methylation of mononitrile [*closo*-B_10_H_9_-1-CN]^2–^ failed and led to a complex mixture of products. The nitrilium **3** was relatively stable under acidic conditions but quickly hydrolyzed upon treatment with base, which eventually led to formation of the carboxyl group.

In a one-pot procedure, nitrile **2[Bu**_**4**_**N]** was reacted with CF_3_SO_3_Me, and the resulting **3** was treated with aqueous NaOH and then HCl to complete the hydrolysis process, giving the desired acid **1e** contaminated with the carbonyl derivative **4** (Scheme 1). The latter was formed by the dehydration of **1e** under acidic conditions. To convert **4** to the acid **1e**, the mixture was treated with NaHCO_3_ to neutralize HCl, and products were extracted and passed through a Dowex ion-exchange column to remove the remaining [Bu_4_N]^+^ counterion. Treatment of the eluate with controlled amounts of [Et_4_N]^+^OH^–^ gave the acid **1e[Et**_**4**_**N]** isolated in 54–59% overall yield.

**Scheme 1:**
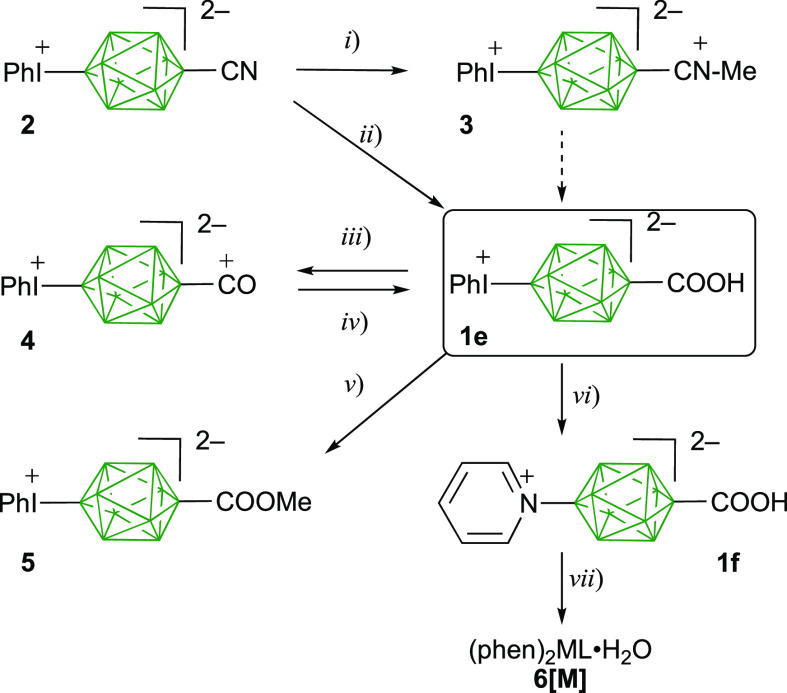
Synthesis of Carboxylic Acid **1e** and Its Derivatives Reagents and conditions: (i) MeOTf, CH_2_Cl_2_, 0–10 °C to rt, 16 h, 68% yield. (ii) One-pot: (1) MeOTf, CH_2_Cl_2_, 0–10 °C to rt, 16 h. (2) Aqueous NaOH, MeCN, 60 °C, 20 min. (3) Concentrated HCl, 10 min. (4) Aqueous NaHCO_3_, CH_2_Cl_2_ extract. (5) MeCN, Dowex. (6) H_2_O, base 1 equiv, 54–59% yield. (iii) MeCN/H_2_O, Dowex; evaporation, 88% yield. (iv) H_2_O, base 1 equiv. (v) (1) MeOH and Dowex. (2) [Et_4_N]^+^OH^–^, 1 equiv, 88% yield. (vi) Pyridine, 85 °C, 16 h, 68–80% yield. (vii) (1) Dowex. (2) NaOH, 2 equiv. (3) M(phen)_2_(NO_3_)_2_·*x*H_2_O, ∼85% yield.

It is remarkable that the labile PhI^+^ group was unaffected during the sequence of transformations of **2[Bu**_**4**_**N]** to **1e[Et**_**4**_**N]**, which includes basic, acidic, and hydrolytic conditions. In contrast, the COOH group in **1e** can be easily esterified. Thus, passing a MeOH solution of **1e[Et**_**4**_**N]** through a Dowex column, followed by neutralization with controlled amounts of [Et_4_N]^+^OH^–^, gave ester **5[Et**_**4**_**N]** in 88% yield (Scheme 1). Passing salt **1e[Et**_**4**_**N]** through Dowex exchange resin, followed by evaporation to dryness, gave essentially pure carbonyl derivative **4** in 88% yield (Scheme 1).

The structures of acid **1e[Et**_**4**_**N]** and intermediate **3** were confirmed with single-crystal XRD analysis (Figure 2). The experimental B–I distances of 2.179(3) and 2.190(6) Å, respectively, are similar to those in the reported^29^ [*closo*-B_10_H_8_-1,10-(PhI)_2_] (avg. 2.178 Å). The B–COOH bond length in **1e[Et**_**4**_**N]** is 1.582(4) Å, which is comparable with 1.573(2) Å in **1d**.^25^ The C≡NMe group in **3** is the most interesting: it is nearly linear [α_CNMe_ = 177.9(6)°] with B–C, C≡N, and N–Me distances of 1.54(1), 1.125(8), and 1.418(8) Å, respectively. A comparison with the structural data for the five closest analogues, *arachno* derivatives containing the B–C≡N–Me fragment,^31−33^ shows that the C≡N and N–Me bonds in **3** are shorter than those reported previously (1.139–1.144 and 1.431–1.436 Å, respectively). Further analysis indicates that methylation of the nitrile little affects the B–C and C≡N distances (1.543(2) and 1.151(2) Å, respectively, in the close analogue [*closo*-B_10_H_8_-1-CN-10-Pyr]^−^).^26^ Also, the height of the tetragonal pyramid [defined as B(1)···B(2)–B(5)] in **3** and its close analogue is within experimental error (1.049 vs 1.052 Å). The structural data suggest that the electron-withdrawing strength of the onium substituents follows the order: Pyr^+^ < SMe_2_^+^ ∼ C≡NMe^+^ < IPh^+^ < N_2_^+^.^26,29^ The acid forms centrosymmetric dimers connected by two close B–I nonbonding interactions (3.625 and 3.675 Å; see the Supporting Information, SI).

**Figure 2:**
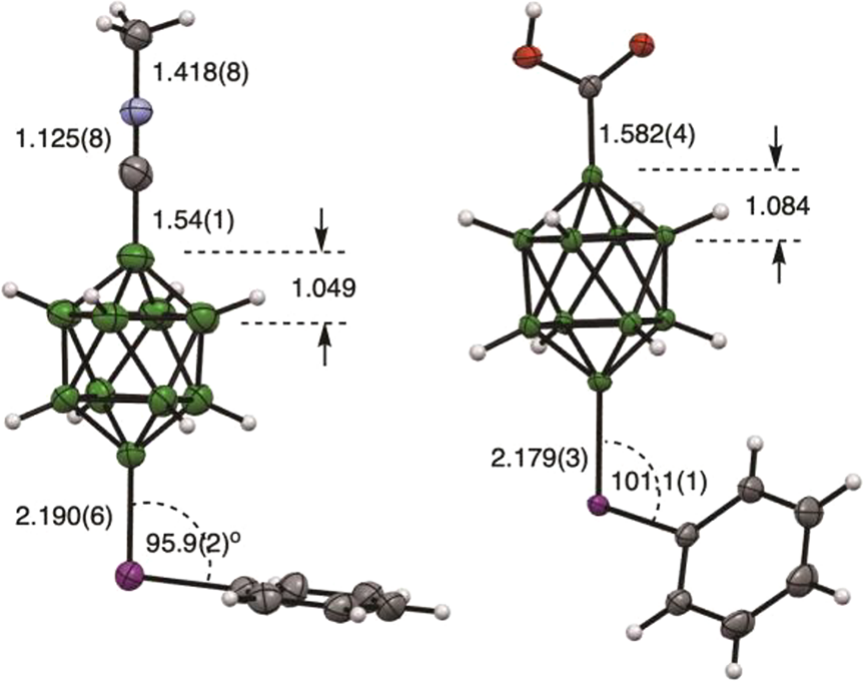
Displacement ellipsoid diagrams for **1e[Bu**_**4**_**N]** (left) and **3** (right) with pertinent geometrical dimensions. For other geometrical parameters, see the text and SI. Thermal ellipsoids are at the 50% probability level. The molecule of solvent and cation are omitted for clarity. Color codes: C, gray; B, green; O, red; I, purple.

The synthetic utility of acid **1e[Et**_**4**_**N]** was demonstrated by replacing PhI with the pyridinium group and the formation of metal complexes. Thus, the reaction of **1e[Et**_**4**_**N]** with pyridine at 85 °C gave the pyridinium derivative **1f[Et**_**4**_**N]**, which was isolated in 68–80% yield (Scheme 1). As in the case of acid **1e**, dehydration of acid **1f** under acidic conditions and formation of the corresponding carbonyl derivative were also observed.

The acid **1f[Et**_**4**_**N]** was transformed to the double sodium salt **6[2Na]** by passing it in MeCN through a Dowex ion-exchange column and treatment of the eluate with NaOH (2 equiv). The subsequent addition of Cu(phen)_2_(NO_3_)_2_·H_2_O^34^ resulted in a precipitate, which was recrystallized from hot MeCN with a few drops of H_2_O, giving green copper(II) complex **6[Cu]** in 84% yield (Scheme 1). Similarly, off-white zinc(II) complex **6[Zn]** was obtained in 86% yield from **6[2Na]** and Zn(phen)_2_(NO_3_)_2_·2H_2_O.^35^ Both complexes were characterized by solution NMR spectroscopy (**6[Zn]**), IR, mass spectrometry, and combustion analysis, demonstrating their expected composition and stoichiometry. IR spectroscopy revealed that the C=O peak of the acid **1f** at *v*_CO_ = 1633 cm^–1^ is absent in the salts **6[Cu]** and **6[Zn]** (see the SI), suggesting that both oxygen atoms of the COO group coordinate to the metal ion. Attempts at obtaining a single crystal suitable for XRD analysis were unsuccessful, and only morphologically homogeneous microcrystalline powders were obtained (see the SI for details).

Electronic absorption spectroscopy revealed a CT band at 330 nm tailing to 400 nm in the carboxylate dianion **6[2Na]** in an MeCN/H_2_O solution and a broad band tailing to 360 nm in solid Zn(phen)_2_(NO_3_)_2_·2H_2_O (Figure 3). Analysis of the solid-state spectrum of complex **6[Zn]** showed features present in the Zn precursor below 350 nm and a broad tailing absorption above 360 nm, presumably related to the cage-to-pyridine CT.

**Figure 3:**
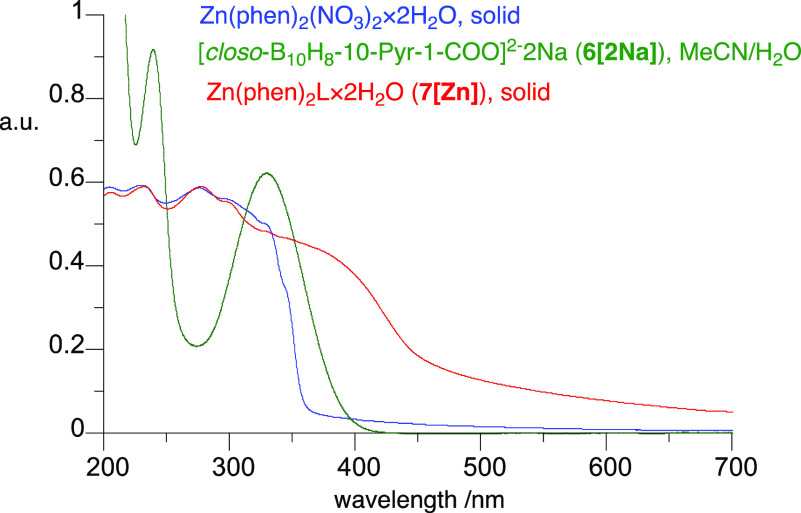
UV–vis solution spectrum of disodium salt **6[2Na]** (green) and solid-state spectra of precursor Zn(phen)_2_(NO_3_)_2_·2H_2_O (blue) and complex **6[Zn]** (red).

In summary, the carboxylic acid **1e[Et**_**4**_**N]**, a versatile intermediate to a potentially broad class of anionic carboxylic acids [*closo*-B_10_H_8_-10-X-1-COOH]^2–^, was obtained in four steps and about 26% overall yield from the parent anion **A**. The potential of the PhI group as a convenient synthetic handle for the introduction of functional groups X was demonstrated by the preparation of photoactive pyridinium acid **1f[Et**_**4**_**N]** and subsequently its complexes **6[M]** with the (phen)_2_Cu^2+^ and (phen)_2_Zn^2+^ ions. The acid exhibits an intramolecular CT band also present in the solid-state **6[Zn]** complex. The readily available intermediate **1e[Et**_**4**_**N]**, and the documented chemistry of the phenyliodonium zwitterions open up access to an unexplored class of functional and possibly polytopic zwitterions (e.g., X = N_3_, SCN, azines) anionic carboxylic acids with applications in metal-ion complexes with balanced charges.

## Supporting Information Available

Synthetic details, copies of NMR spectra, XRD data collection details, and UV–vis data (PDF)
