# Supplementary material for: [closo-B10H8-10-PhI-1-COOH]− Anion: An Intermediate for Functional Anionic Carboxylate Ligands
Source: Inorg Chem. 2024 Jul 12;63(30):13831–4. doi: 10.1021/acs.inorgchem.4c02044 (PMC11289747; doi:10.1021/acs.inorgchem.4c02044)
Supplement: Supplementary file 1 — ic4c02044_si_001.pdf [file ic4c02044_si_001.pdf]

Supporting Information

[*closo*-$B_{10}H_8$-10-PhI-1-COOH]$^-$ anion: An intermediate for functional anionic carboxylate ligands

Rafał Jakubowski,[a,b] Szymon Kapuściński,[a,b] Oleksandr Hietsoi,[a] Andrienne C. Friedli,[a] and Piotr Kaszyński*[a,b,c]

[a] Department of Chemistry, Middle Tennessee State University, Murfreesboro, TN 37132, USA
[b] Centre for Molecular and Macromolecular Studies, Polish Academy of Sciences, Sienkiewicza 112, 90-363 Łódź, Poland

[c] Faculty of Chemistry, University of Łódź, Tamka 12, 91-403 Łódź, Poland

Table of Content:

1. Synthetic details S2

2. NMR spectra S8

3. IR spectra S19

4. UV-vis absorption spectroscopy S20

5. XRD data collection and refinement S20

6. Attempts at growing crystals of metal complexes **6[M]** S25

7. References S26

1. Synthetic details

General. Reagents and solvents were obtained commercially. Anion [*closo*-$B_{10}H_{10}$]$^{2-}$ was obtained from $B_{10}H_{14}$ according to a literature procedure.[1] Reactions were conducted in argon and subsequent manipulations were carried out in air. Column chromatography was performed using 70–230 mesh silica gel (Merck). Uncorrected melting points were recorded in capillary tubes. NMR spectra were obtained at 500 MHz (^{1}H), 126 MHz (^{13}C) and 160 MHz (^{11}B) or at 400 MHz (^{1}H), 101 MHz (^{13}C) and 128 MHz (^{11}B) in acetone-*d*$_6$, D_2O (**6[2Na]**) or CD_3CN (**6[Zn]** and **1f[H$_3$O]**). Chemical shifts were referenced to the solvent (acetone-*d*$_6$: 2.05 ppm for ^{1}H and 29.84 ppm for ^{13}C; CD_3CN 1.94 ppm for ^{1}H and 118.26 ppm for ^{13}C; D_2O 4.79 ppm for ^{1}H)[2] and to an external sample of neat BF_3•Et_2O in acetone-*d*$_6$ or CD_3CN (^{11}B, δ = 0.0 ppm). ^{11}B NMR chemical shifts were taken from the H-decoupled spectra. IR spectra were recorded for neat samples using an ATR attachment. HR mass spectrometry was conducted with the TOF-MS ES method, most often in the negative mode.

Ion exchange resin for cation exchange. Dowex-50 was soaked in H_2O and decanted (3x). It was then poured onto column, washed with small amount of MeCN and used as a stationary phase for ion exchange chromatography.

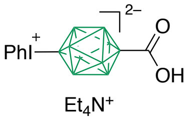

One-pot procedure for the preparation of [*closo*-B$_{10}$H$_8$-10-IPh-1-COOH]$^-$ [Et$_4$N]$^+$ (1e[Et$_4$N]). [*closo*-$B_{10}H_8$-10-IPh-1-CN]$^-$ [Bu_4N]$^+$ (ref.[3]; **2[Bu$_4$N]**, 600.0 mg, 1.019 mmol) was dissolved in dry CH_2Cl_2 (9 mL) and MeOTf (234 mg, 156 µL, 1.147 mmol, 1.4 eq) was added dropwise at 0 °C under argon. The reaction mixture was stirred at 0 °C for 1 h, allowed to reach rt and stirring was continued overnight. After 20 h, the starting material was completely consumed, giving the methylated adduct [*closo*-$B_{10}H_8$-10-IPh-1-CNMe] (**3**) as the only product based on ^{11}B NMR. The reaction mixture was evaporated to dryness, the residue was dissolved in MeCN (3 mL), aqueous NaOH (68. mg, 1.38 mmol, 1.68 eq) in 3 mL of H_2O was added, and the mixture stirred at 60 °C until homogeneous (typically 20 min), then MeCN was evaporated under vacuum to form a slurry. Conc. aq. HCl (250 µL, ~2.5 mmol) was added

and the reaction mixture was stirred for 10 min, resulting in the formation of a mixture of the carboxylic acid **1e** and the carbonyl derivative **4**. Aq. $NaHCO_3$ was added to neutralize excess HCl, and the products were extracted with CH_2Cl_2 (3 x 10 mL). The solvent was evaporated giving a mixture of the carboxylic acid **1e[Bu$_4$N]** and, most probably, its carboxylate derivative (based on ^{11}B NMR spectrum). The crude mixture was dissolved in MeCN and passed through a Dowex-40W ion exchange resin. Water was added to the eluate and MeCN was evaporated. The resulting cloudy mixture was treated with diluted aqueous $[Et_4N]^+[OH]^-$ until slightly basic pH (monitored with a pH paper). During basification a white precipitate started to form. It was filtered off, washed with cold H_2O and dried giving 195 mg (59% yield) of pure carboxylic acid **1e[Et$_4$N]** as colorless crystals. The filtrate was extracted with CH_2Cl_2 (3 x 10 mL) and evaporated giving additional 78 mg of a mixture of the carboxylic acid **1e[Et$_4$N]** and the carbonyl derivative **4** as a greenish powder. Acid **1e[Et$_4$N]** can be recrystallized from H_2O/MeCN or from EtOAc/CH_2Cl_2: mp 158 °C; ^{1}H NMR (500 MHz, acetone-*d*$_6$) δ 0.25 – 1.27 (br m, 8H), 1.37 (tt, J_1 = 7.3 Hz, J_2 = 1.9 Hz, 12H), 3.47 (q, J = 7.3 Hz, 8H), 7.46 (t, J = 7.9 Hz, 2H), 7.61 (tt, J_1 = 7.4 Hz, J_2 = 1.0 Hz, 1H), 8.20 (dd, J_1 = 8.5 Hz, J_2 = 0.9 Hz, 2H), 8.36 (br s, 1H); ^{13}C NMR (126 MHz, acetone-*d*$_6$) δ 7.7, 53.0, 104.6, 131.3, 132.1, 135.5; ^{11}B NMR (160 MHz, acetone-d$_6$) δ -24.9 (d, J = 135 Hz, 4B), -23.2 (d, J = 141 Hz, 4B), 1.7 (s, 1B), 10.9 (s, 1B); IR (ATR) *v* 2993 (OH), 2482 (BH), 1631 (C=O), 1475, 1277, 994, 741cm^{-1}; HRMS (ESI-) *m/z* calcd. for $C_7H_{14}B_{10}IO_2$ $[M]^+$: 367.0969, found: 367.0960. Anal. Calcd. for $C_{15}H_{34}B_{10}INO_2$: C, 36.36; H, 6.92; N, 2.83. Found: C, 36.42; H, 6.75; N, 2.80.

Note: washing with more basic aq Na_2CO_3 instead of $NaHCO_3$ leads to the formation of a mixture of carboxylic acid and its sodium salt.

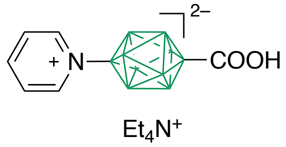

Preparation of [*closo*-B$_{10}$H$_8$-10-Pyr-1-COOH]$^-$[Et$_4$N]$^+$ (1f[Et$_4$N]). Dry pyridine (2.5 mL) was added to acid **1e[Et$_4$N]** (49.5 mg, 0.100 mmol) and the reaction mixture was stirred overnight at 85 °C under argon. Pyridine was removed in high vacuum and the residue was washed with warm hexane (2x) and co-evaporated with toluene (2x) resulting in formation of a mixture of acid **1f** and, presumably, carbonyl derivative [*closo*-B$_{10}$H$_8$-10-Pyr-1-CO], based on ^{11}B NMR. To remove the byproduct, the crude mixture was stirred overnight with

CH_2Cl_2 (3 mL) at rt, diluted with hexane (1 mL) and placed in a refrigerator for 1 hr. The solids were filtered off, and were recrystallized from MeCN/H_2O to give 25 mg (68% yield) of pure product **1f[Et$_4$N]** as a yellow solid: mp 135 °C (decomp); ^{1}H NMR (400 MHz, acetone-d_6) δ 0.15 – 1.95 (m, 8H), 1.38 (tt, J_1 = 7.3, J_2 = 1.8 Hz, 12H), 3.48 (q, J = 7.3 Hz, 8H), 7.98 (t, J = 7.1 Hz, 2H), 8.29 (bs, 1H), 8.43 (t, J = 7.8 Hz, 1H), 9.53 (d, J = 5.4 Hz, 2H); ^{13}C NMR (101 MHz, acetone-d_6) δ 7.7, 53.0 (t, J = 3.0 Hz), 126.9, 142.4, 148.7; ^{11}B NMR (128 MHz, acetone-d_6) δ -25.4 (d, J = 144 Hz, 4B), -23.6 (d, J = 138 Hz, 4B), 5.1 (s, 1B), 17.5 (s, 1B); IR (ATR) v 2987, 2471 (BH), 1634 (C=O), 1460, 998, 694 cm^{-1}; HRMS (ESI-) *m/z* calcd. for $C_6H_{14}B_{10}NO_2$ [M]$^+$: 242.1955, found: 242.1965. Anal. Calcd. for $C_{14}H_{34}B_{10}N_2O_2$: C, 45.38; H, 9.25; N, 7.56. Found: C, 45.29; H, 8.97; N, 7.31.

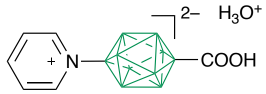

Preparation of [*closo*-$B_{10}H_8$-10-Pyr-1-COOH]$^-$[H_3O]$^+$ (1f[H_3O]). [*closo*-$B_{10}H_8$-1-COOH-10-C_5H_5N]$^-$[Et_4N]$^+$ (**1f[Et$_4$N]**, 10.0 mg, 0.027 mmol) was dissolved in MeCN (1 mL) and passed through Dowex-50 ion exchange resin and the eluate was evaporated to dryness. ^{1}H NMR (400 MHz, D_2O/CD_3CN) δ 0.0 – 1.65 (m, 8H), 7.93 (t, J = 7.0 Hz, 2H), 8.41 (t, J_1 = 7.8 Hz, 1H), 9.45 (d, J = 5.5 Hz, 2H); ^{11}B NMR (128 MHz, D_2O/CD_3CN) δ -27.6 (d, J = 131 Hz, 4B), -26.3 (d, J = 126 Hz, 4B), 1.7 (s, 1B), 18.0 (s, 1B); UV (MeCN/H_2O) λ_{max} 207.5, 239, 330 nm.

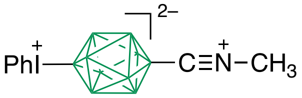

Preparation of [*closo*-$B_{10}H_8$-10-IPh-1-CNMe] (3). To a solution of [*closo*-$B_{10}H_8$-10-IPh-1-CN]$^-$[Et_4N]$^+$ (**2[Et$_4$N]**, 100 mg, 0.277 mmol) in dry CH_2Cl_2 (2 mL), MeOTf (42.3 µL, 0.388 mmol, 1.4 eq) was added dropwise at 10 °C under argon atmosphere and stirred for 10 min at 10 °C, then at rt overnight. Once all starting material was consumed (by ^{11}B NMR), the reaction mixture was washed with water (2x) and the organic layer was evaporated. The crude product was recrystallized by slow evaporation of EtOH/CH_2Cl_2 to give 68.0 mg (68% yield) of pure **3** as grayish shiny crystals: mp darken at 160 °C without melting below 400 °C; ^{1}H NMR (500 MHz, acetone-d_6) δ 1.07 (br q, J = 129 Hz, 8H), 4.13 (t, J = 2.4 Hz, 3H), 7.50 (t, J = 7.9 Hz, 2H), 7.66 (t, J = 7.5 Hz, 1H), 8.18 (dd, J_1 = 8.4 Hz, J_2 = 0.9 Hz, 2H); ^{13}C NMR

(126 MHz, acetone-d_6) δ 31.1 (t, J = 7.9 Hz), 105.3, 128.5 (br s), 131.7, 132.3, 136.2; ^{11}B NMR (160 MHz, acetone-d_6) δ -21.3 (d, J = 142 Hz, 4B), -20.4 (d, J = 141 Hz, 4B), -7.9 (s, 1B), 9.8 (s, 1B); IR (ATR) *v* 2486 (BH), 2296 (CNMe), 992, 741 cm^{-1}; HRMS (AP-) *m/z* calcd. for $C_8H_{13}B_{10}IN$ [M-3]$^+$: 360.1023, found: 360.1004. Anal. Calcd. for $C_8H_{16}B_{10}IN$: C, 26.60; H, 4.46; N, 3.88. Found: C, 26.73; H, 4.39; N, 3.62.

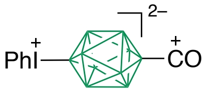

[*closo*-B$_{10}$H$_8$-10-IPh-1-CO] (4). A solution of [*closo*-B$_{10}$H$_8$-10-IPh-1-COOH]$^-$[Et$_4$N]$^+$ (**1e[Et$_4$N]**, 280 mg, 0.565 mmol) in MeCN/H_2O was passed through Dowex-50 ion exchange column and the eluate was evaporated leaving 174 mg (88% yield) of the essentially pure carbonyl derivative **4** as a white powder: ^{1}H NMR (500 MHz, acetone-d_6) δ 1.32 (br q, J = 143.7 Hz, 4H), 1.52 (br q, J = 147.6 Hz, 4H), 7.53 (t, J = 7.9 Hz, 2H), 7.69 (t, J = 7.4 Hz, 1H), 8.19 (d, J = 8.1 Hz, 2H); ^{13}C NMR (126 MHz, acetone-d_6) δ 106.2, 132.1, 132.5, 136.7, 175.0 (m, low int, CO); ^{11}B NMR (160 MHz, acetone-d_6) δ -20.0 (s, 1B), -18.5 (d, J = 141 Hz, 4B), -14.4 (d, J = 148 Hz, 4B), 18.8 (s, 1B); IR (ATR) *v* 2516 (BH), 2132 (CO), 1473, 1194, 992, 736 cm^{-1}.

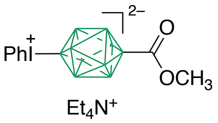

Preparation of [*closo*-B$_{10}$H$_8$-10-IPh-1-COOMe]$^-$[Et$_4$N]$^+$ (5[Et$_4$N]). A solution of acid **1d[Et$_4$N]** (280 mg, 0.565 mmol) in MeOH was passed through Dowex-50 ion exchange resin and the eluate was treated with 35% aqueous [Et$_4$N]$^+$[OH]$^-$ until slightly basic pH (monitored with paper indicator). The solution was concentrated and left overnight for product precipitation. The precipitate was crystallized from MeOH by slow evaporation of solvent to give 252 mg (88% yield) of pure product as a white powder: mp 154 – 155 °C; ^{1}H NMR (500 MHz, acetone-d_6) δ 0.40 – 1.19 (br m, 8H), 1.37 (tt, J_1 = 7.2 Hz, J_2 = 1.9 Hz, 12H), 3.46 (q, J = 7.3 Hz, 8H), 3.58 (s, 3H), 7.46 (t, J = 7.9 Hz, 2H), 7.60 (tt, J_1 = 7.4 Hz, J_2 = 1.1 Hz, 1H), 8.21 (d, J = 8.0 Hz, 2H); ^{13}C NMR (126 MHz, acetone-d_6) δ 7.7, 48.0, 53.0, 104.5, 131.2, 132.0, 135.4 (C=O not observed); ^{11}B NMR (160 MHz, acetone-d_6) δ -24.8 (d, J = 136 Hz, 4B), -23.1 (d, J = 136 Hz, 4B), 1.3 (s, 1B), 11.8 (s, 1B); IR (ATR) *v* 2989, 2495 (BH), 1674 (C=O), 1473, 1236,

1056, 991, 743 cm^{-1}; HRMS (ESI-) *m/z* calcd. for $C_8H_{16}B_{10}IO_2$ $[M]^+$: 381.1126, found: 381.1113. Anal. Calcd. for $C_{16}H_{36}B_{10}INO_2$: C, 37.72; H, 7.12; N, 2.75. Found: C, 37.45; H, 6.85; N, 3.10.

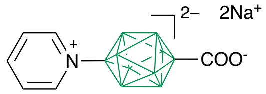

Preparation of [*closo*-$B_{10}H_8$-10-Pyr-1-COO]$^{2-}$2Na^+ (6[2Na]). [*closo*-$B_{10}H_8$-1-COOH-10-C_5H_5N]$^-$[Et_4N]$^+$ (**1f[Et_4N]**, 10.0 mg, 0.027 mmol) was dissolved in MeCN (1 mL) and passed through Dowex-50 ion exchange resin. H_2O (1 mL) was added to the eluate and MeCN was evaporated. NaOH (2.2 mg, 0.054 mmol) was dissolved in H_2O (0.5 mL) was added and the mixture was evaporated to dryness. ^1H NMR (400 MHz, D_2O) δ -0.10 – 1.50 (m, 8H), 7.89 (dd, J_1 = 7.7 Hz, J_2 = 6.8 Hz, 2H), 8.37 (tt, J_1 = 7.8 Hz, J_2 = 1.5 Hz, 1H), 9.44 (d, J = 5.3 Hz, 2H); ^{11}B NMR (128 MHz, D_2O) δ -28.9 (d, J = 135 Hz, 4B), -27.0 (d, J = 129 Hz, 4B), 6.0 (s, 1B), 14.6 (s, 1B); UV (MeCN/H_2O) λ_{max} 208, 240, 330 nm.

Preparation of [10-C_5H_5N-*closo*-$B_{10}H_8$-1-COO]Cu(phen)$_2$ (6[Cu]). [*closo*-$B_{10}H_8$-1-COOH-10-C_5H_5N]$^-$[Et_4N]$^+$ (**1f[Et_4N]**, 10.0 mg, 0.027 mmol) was dissolved in MeCN (1 mL) and passed through Dowex-50 ion exchange resin. H_2O (1 mL) was added to the eluate and MeCN was evaporated. NaOH (2.2 mg, 0.054 mmol) was dissolved in H_2O (0.5 mL) was added and the mixture was evaporated to dryness. Complex Cu(phen)$_2$(NO_3)$_2$•H_2O[4] (15.3 mg, 0.027 mmol) was dissolved on heating in MeCN/H_2O mixture (1 mL, 1:1, *v/v*) and [*closo*-$B_{10}H_8$-1-COO-10-C_5H_5N]•2Na was added to this solution immediately forming a green solid. It was evaporated to dryness and dissolved in hot MeCN (2 mL). The insoluble solids were filtered off. A few drops of H_2O were added to the remaining MeCN solution and it was left for several days at 4 °C for slow crystallization to give 15.0 mg (84% yield) of the final complex as green crystals: IR (ATR) *v* 3381, 3064, 2465 (BH), 1622, 1426, 843, 684 cm^{-1}; HRMS (ESI+) *m/z* calcd. for $C_{30}H_{30}B_{10}N_5O_2Cu$ $[M+1]^+$: 665.2626, found: 665.2614. Anal. Calcd. for $C_{30}H_{29}B_{10}N_5O_2Cu$: C, 54.33; H, 4.41; N, 10.56. Calcd. for $C_{30}H_{29}B_{10}N_5O_2Cu$•2$H_2O$: C, 51.53; H, 4.76; N, 10.02. Found: C, 50.97; H, 4.22; N, 10.34.

Preparation of [10-C_5H_5N-*closo*-$B_{10}H_8$-1-COO]Zn(phen)$_2$ (6[Zn]). [*closo*-$B_{10}H_8$-1-COOH-10-C_5H_5N]$^-$[Et_4N]$^+$ (**1f[Et_4N]**, 10.0 mg, 0.027 mmol) was dissolved in MeCN (1 mL) and passed through Dowex-50 ion exchange resin. H_2O (1 mL) was added to the eluate and MeCN was

evaporated. NaOH (2.2 mg, 0.054 mmol) was dissolved in H_2O (0.5 mL) was added and the mixture was evaporated to dryness. $Zn(phen)_2(NO_3)_2$•$2H_2O$ (ref [5], 15.8 mg, 0.027 mmol) was dissolved on heating in MeCN/H_2O mixture (2 mL, 1:1, *v/v*) and [*closo*-$B_{10}H_8$-1-COO-10-C_5H_5N]•2Na (**6[2Na]**) was added to this solution immediately forming a pale-yellow solid. MeCN was evaporated, the resultant mixture was cooled down and the solids were filtered off. The solids were washed with H_2O (3x) and dried giving 15.4 mg (86% yield) of the final complex as an off-white solid, which was recrystallized from hot MeCN with a few drops of H_2O: ^{1}H NMR (400 MHz, CD_3CN) δ 0.0 – 1.60 (m, 8H), 7.55-7.95 (m, 6H), 7.95 - 8.40 (m, 9H), 8.48-8.93 (m, 5H), 9.45 (d, *J* = 5.4 Hz, 1H); ^{13}C NMR (126 MHz, CD_3CN) δ 126.7, 127.1, 128.6, 131.0, 141.5, 142.8, 148.7, 150.0; ^{11}B NMR (101 MHz, CD_3CN) δ -26.0 (d, *J* = 149 Hz, 4B), -24.4 (d, *J* = 145 Hz, 4B), 4.1 (s, 1B), 17.3 (s, 1B); IR (ATR) *ν* 3389, 3061, 2471 (BH), 1624, 1331, 866, 733 cm^{-1}; HRMS (ESI+) *m/z* calcd. for $C_{30}H_{30}B_{10}N_5O_2Zn$ $[M+1]^+$: 666.2622, found: 666.2638. Anal. Calcd. for $C_{30}H_{29}B_{10}N_5O_2Zn$: C, 54.18; H, 4.40; N, 10.53. Calcd. for $C_{30}H_{29}B_{10}N_5O_2Zn$•$H_2O$: C, 52.75; H, 4.57; N, 10.25. Found: C, 53.19; H, 3.68; N, 11.72.

2. NMR spectra

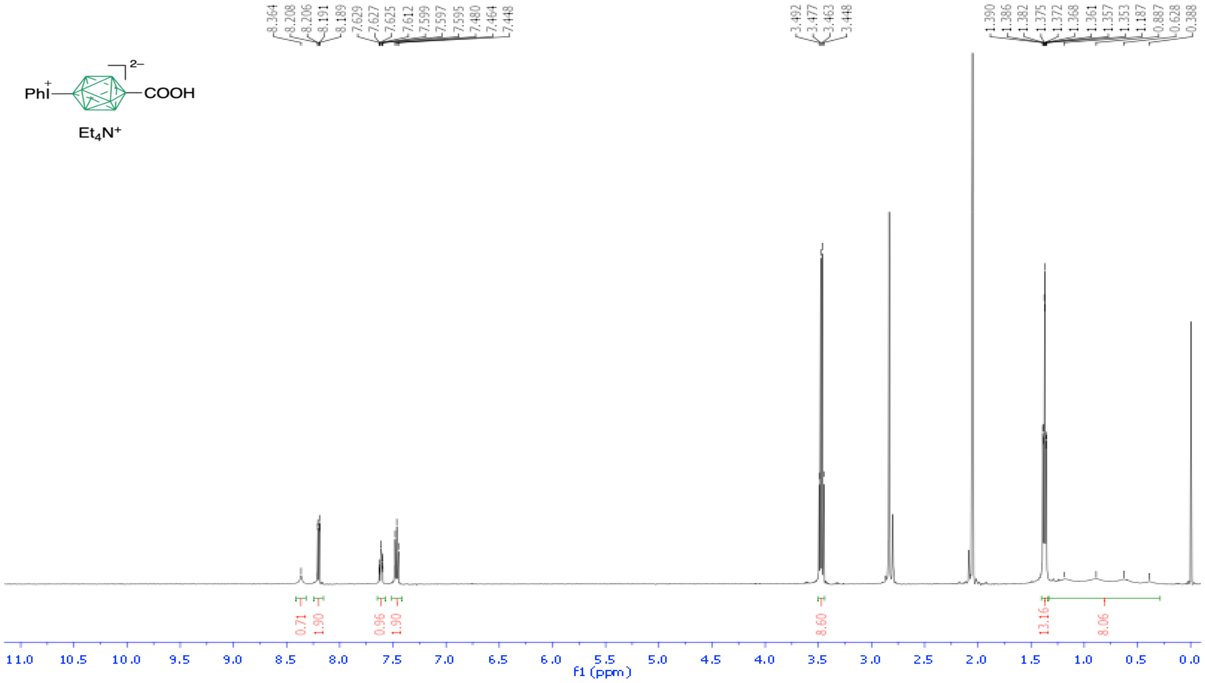

Figure S1. ^{1}H NMR spectrum for [*closo*-$B_{10}H_8$-10-IPh-1-COOH]$^{-}$[Et_4N]$^{+}$ (**1e[Et₄N]**) recorded in acetone-d_6 at 500 MHz.

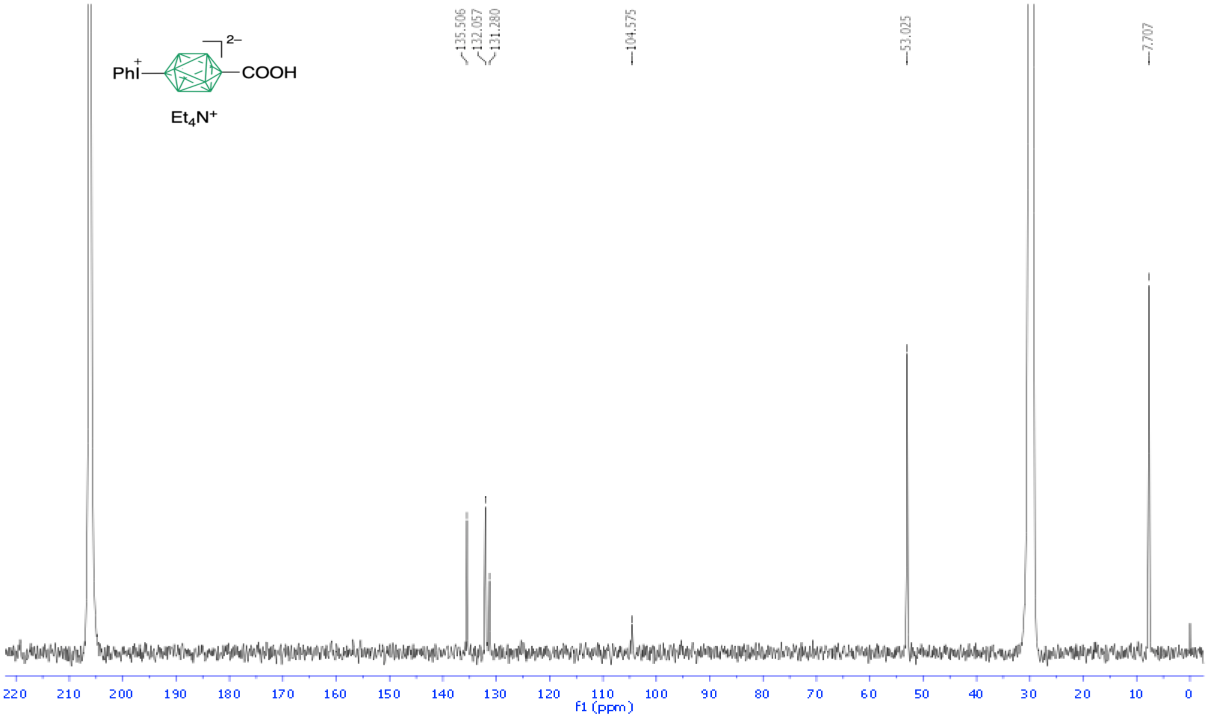

Figure S2. ^{13}C{^{1}H} NMR spectrum for [*closo*-$B_{10}H_8$-10-IPh-1-COOH]$^{-}$[Et_4N]$^{+}$ (**1e[Et₄N]**) recorded in acetone-d_6 at 126 MHz.

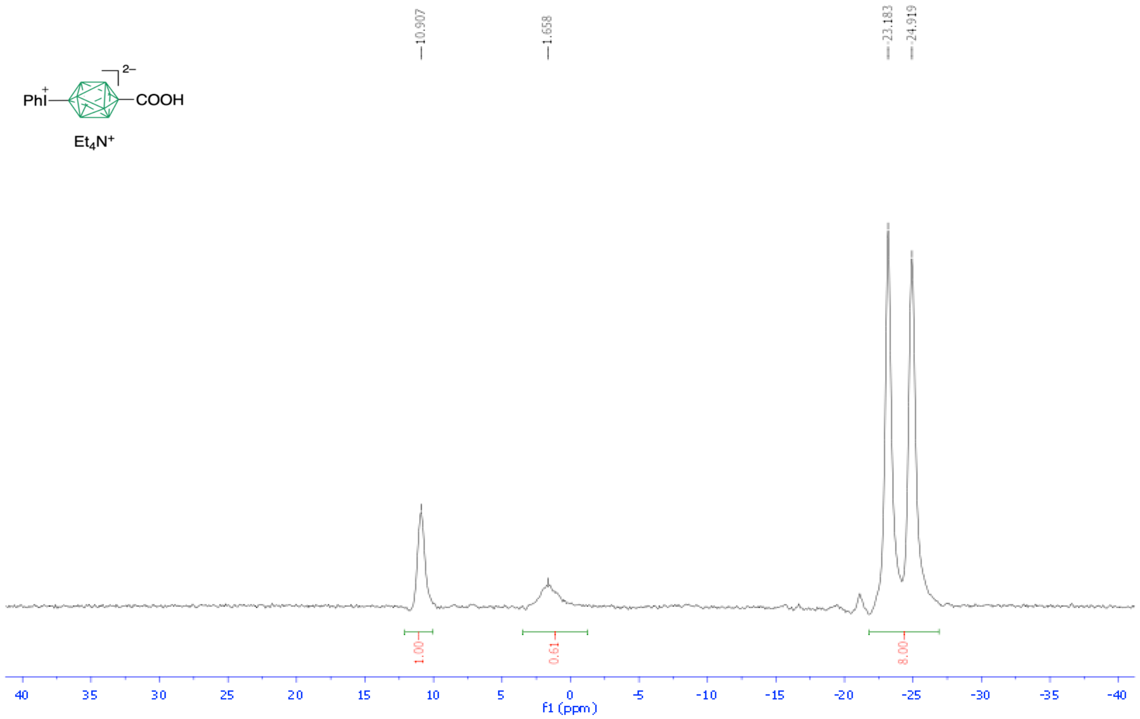

Figure S3. $^{11}B\{^{1}H\}$ NMR spectrum for [*closo*-$B_{10}H_8$-10-IPh-1-COOH]$^-$[Et_4N]$^+$ (**1e[Et₄N]**) recorded in acetone-d_6 at 160 MHz.

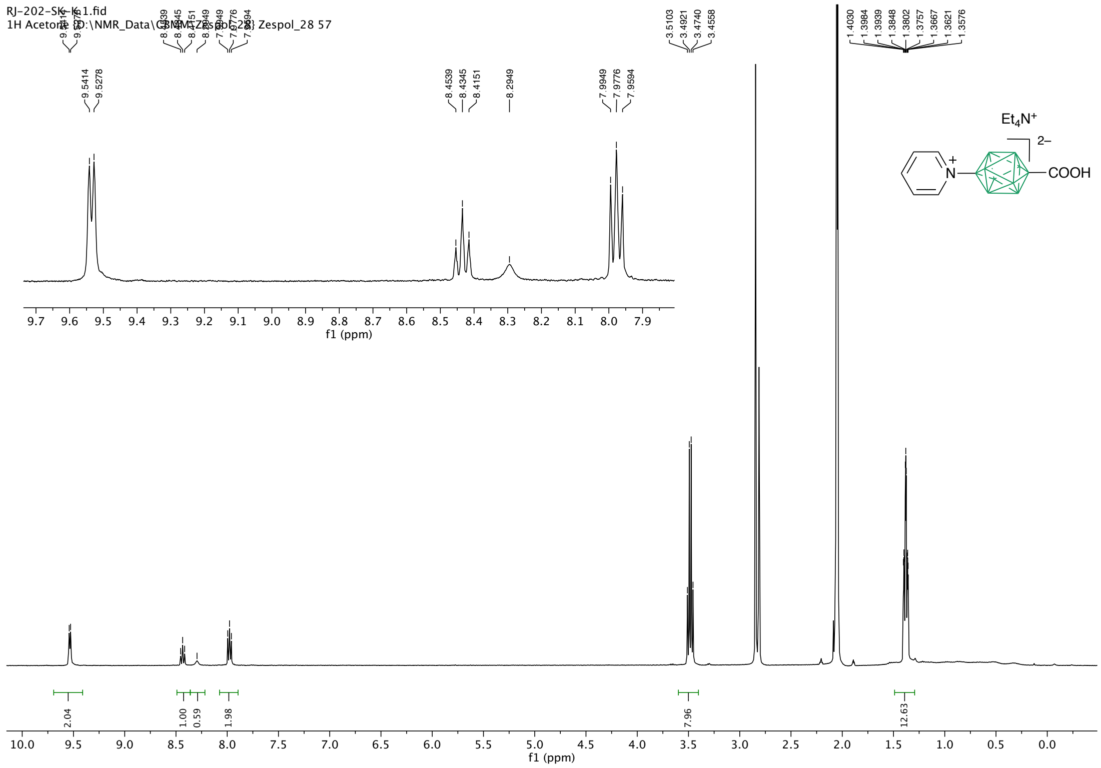

Figure S4. ^{1}H NMR spectrum for [*closo*-$B_{10}H_8$-10-Pyr-1-COOH]$^-$[Et_4N]$^+$ (**1f[Et₄N]**) recorded in acetone-d_6 at 400 MHz.

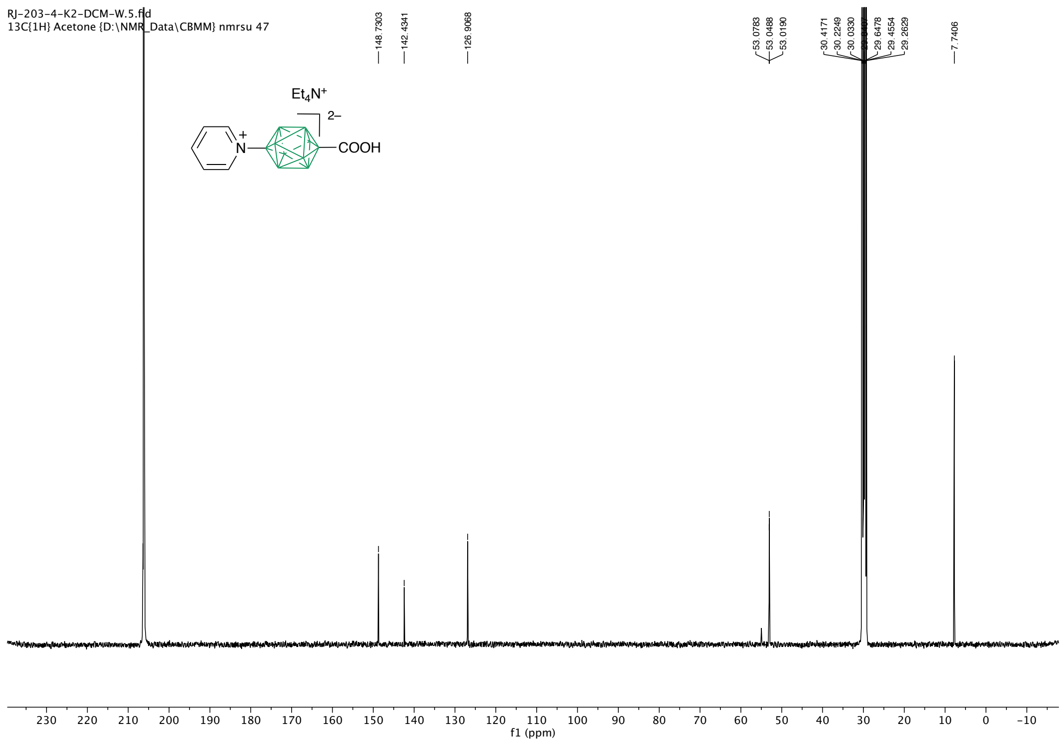

Figure S5. $^{13}C\{^{1}H\}$ NMR spectrum for [*closo*-$B_{10}H_8$-10-Pyr-1-COOH]$^{-}$[Et_4N]$^{+}$ (**1f[Et$_4$N]**) recorded in acetone-d_6 at 101 MHz.

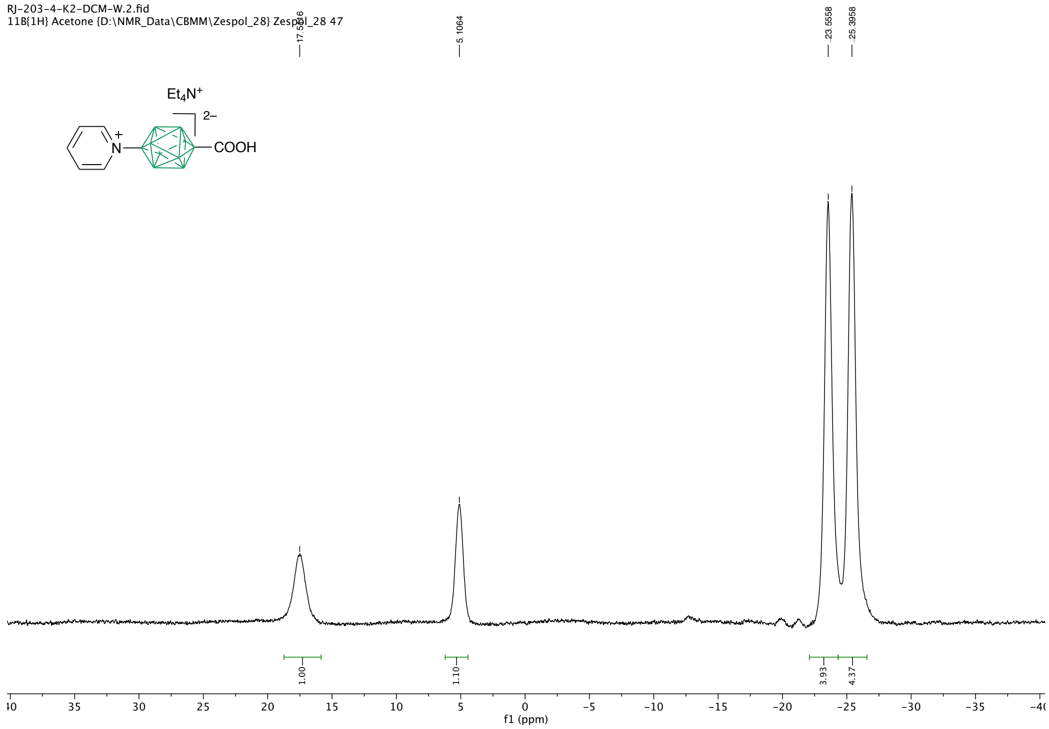

Figure S6. $^{11}B\{^{1}H\}$ NMR spectrum for [*closo*-$B_{10}H_8$-10-Pyr-1-COOH]$^{-}$[Et_4N]$^{+}$ (**1f[Et$_4$N]**) recorded in acetone-d_6 at 160 MHz.

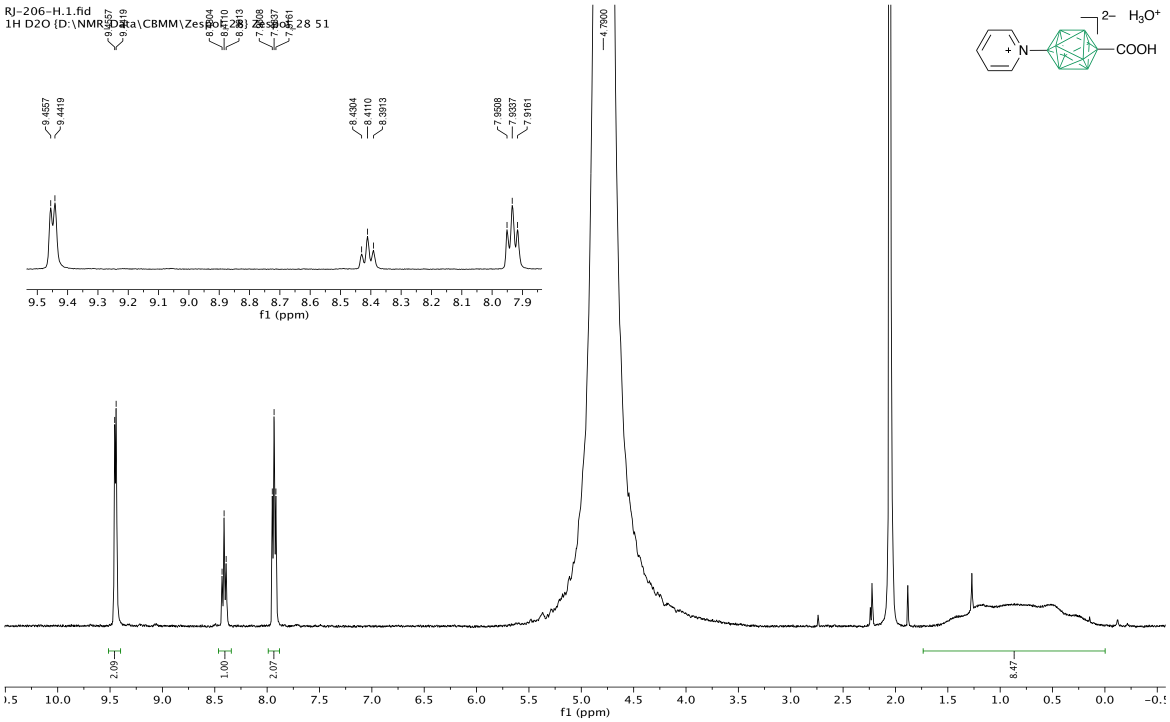

Figure S7. ^{1}H NMR spectrum for crude [*closo*-$B_{10}H_8$-10-C_5H_5N-1-COOH]$^{-}$[H_3O]$^{+}$ (**1f[H₃O]**) recorded in D_2O/CD_3CN mixture (4:1, *v/v*) at 400 MHz.

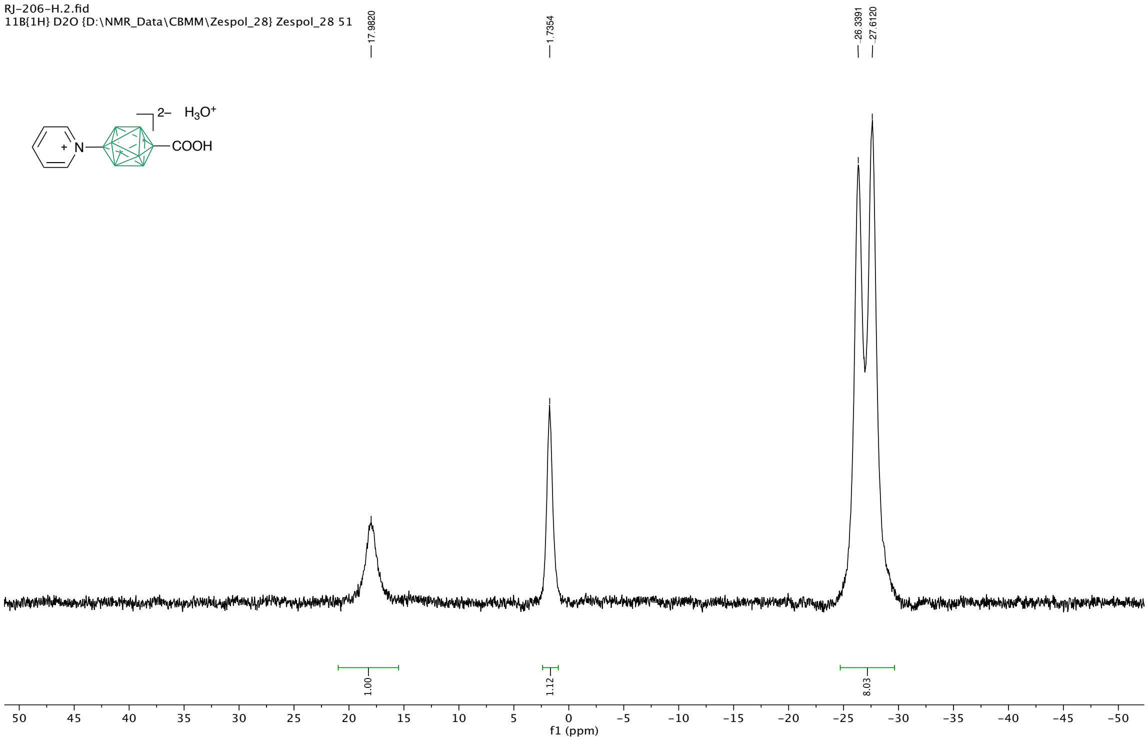

Figure S8. $^{11}B\{^{1}H\}$ NMR spectrum for crude [*closo*-$B_{10}H_8$-10-C_5H_5N-1-COOH]$^{-}$[H_3O]$^{+}$ (**1f[H₃O]**) recorded in D_2O/CD_3CN mixture (4:1, *v/v*) at 128 MHz.

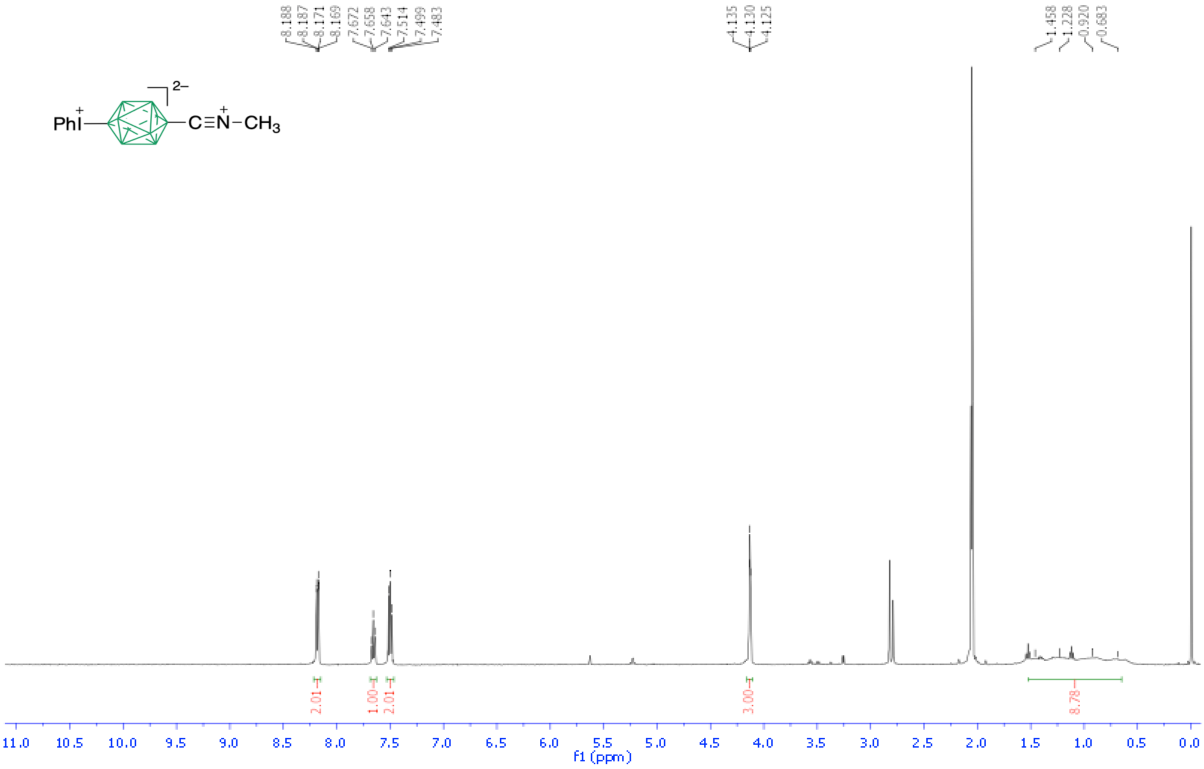

Figure S9. ^{1}H NMR spectrum for [*closo*-$B_{10}H_8$-10-IPh-1-CNMe] (**3**) recorded in acetone-d_6 at 500 MHz.

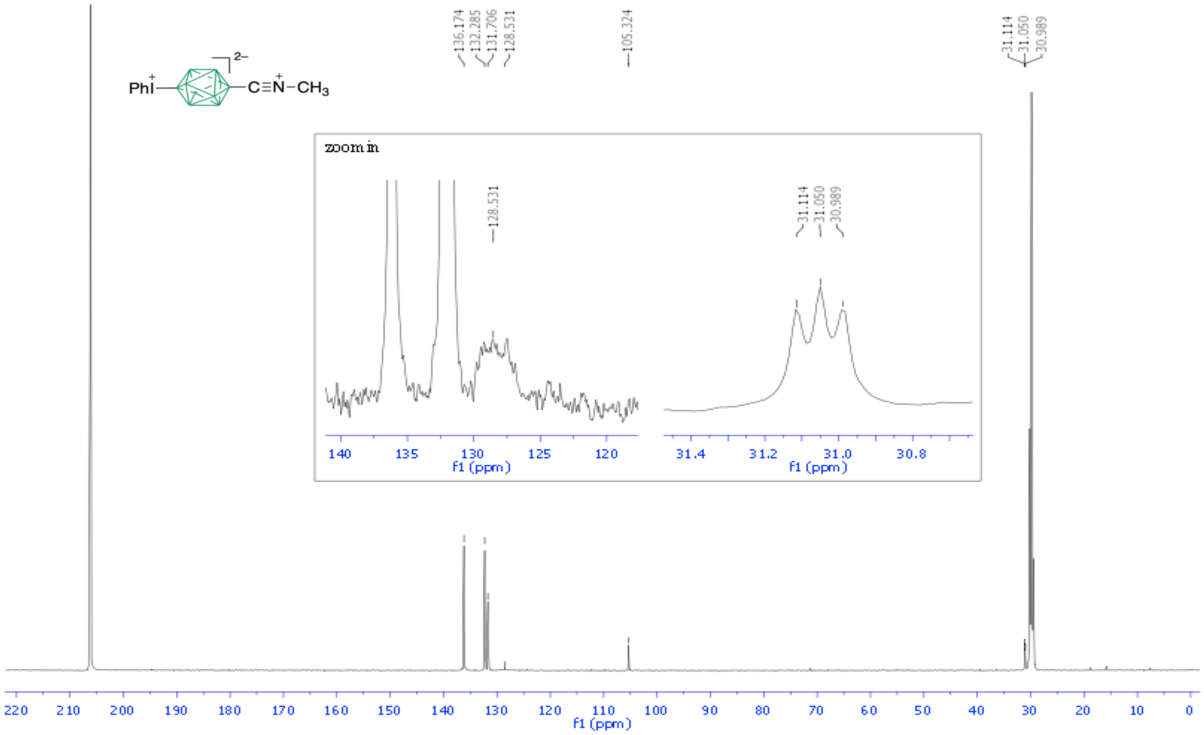

Figure S10. $^{13}C\{^{1}H\}$ NMR spectrum for [*closo*-$B_{10}H_8$-10-IPh-1-CNMe] (**3**) recorded in acetone-d_6 at 126 MHz.

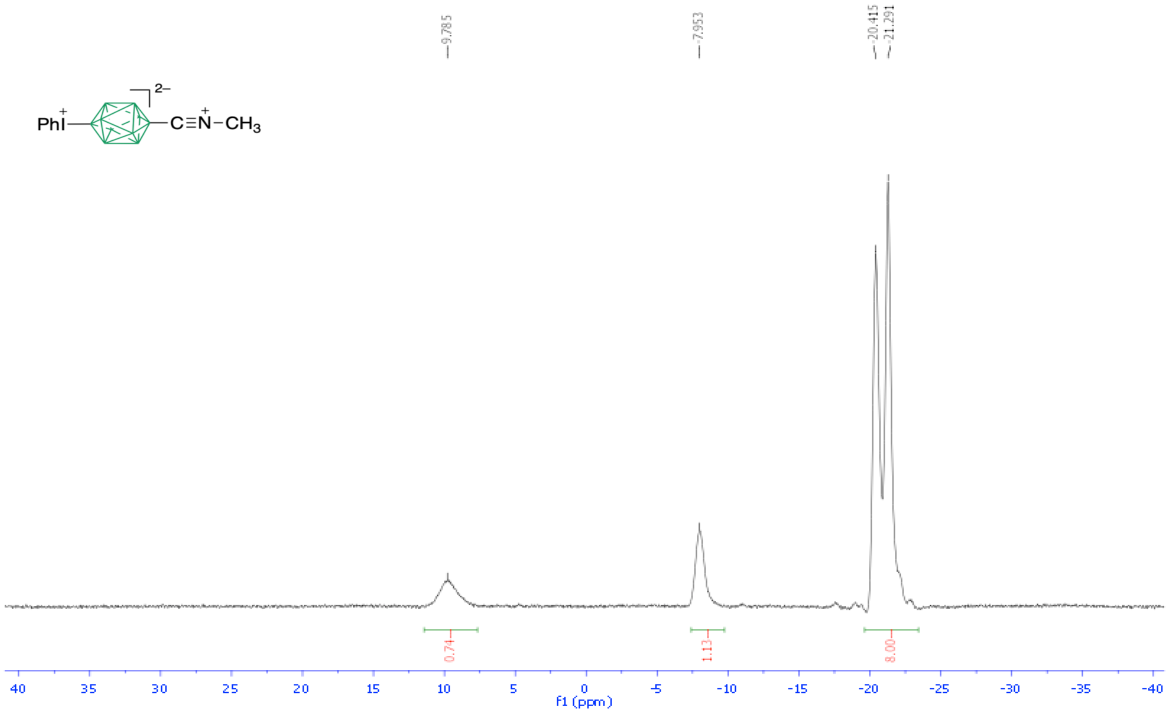

Figure S11. $^{11}B\{^{1}H\}$ NMR spectrum for [*closo*-$B_{10}H_8$-10-IPh-1-CNMe] (**3**) recorded in acetone-d_6 at 160 MHz.

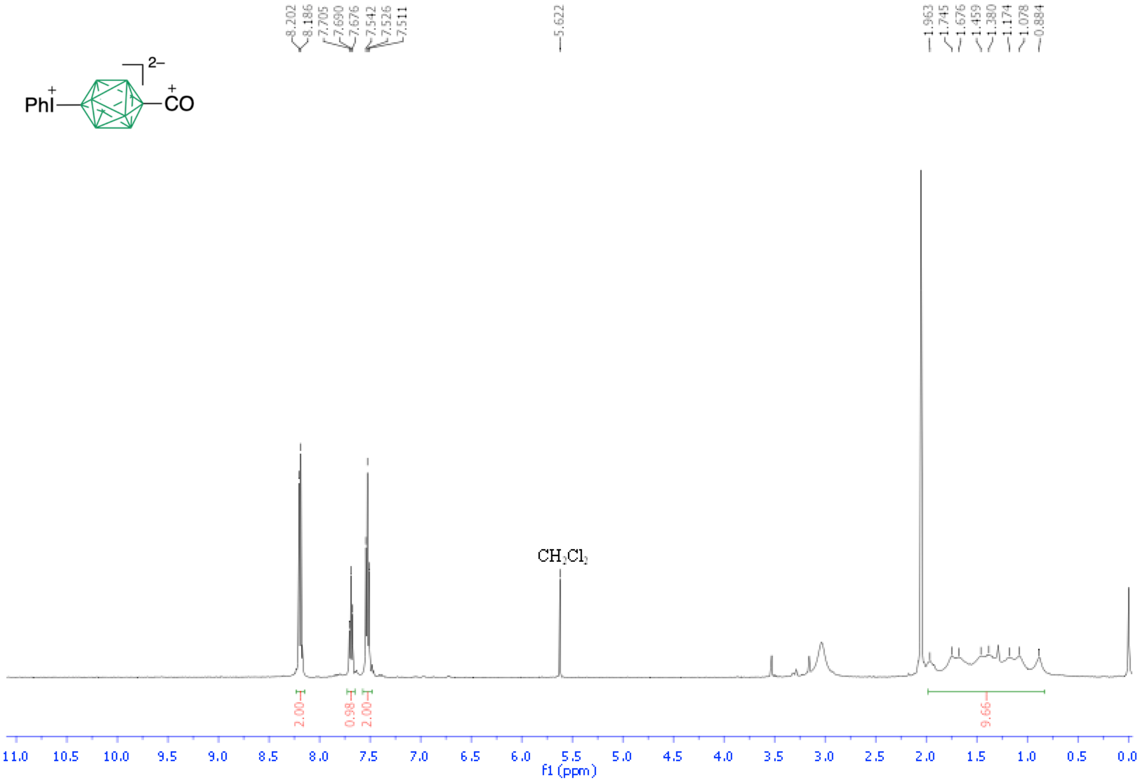

Figure S12. ^{1}H NMR spectrum for crude [*closo*-$B_{10}H_8$-10-IPh-1-CO] (**4**) recorded in acetone-d_6 at 500 MHz.

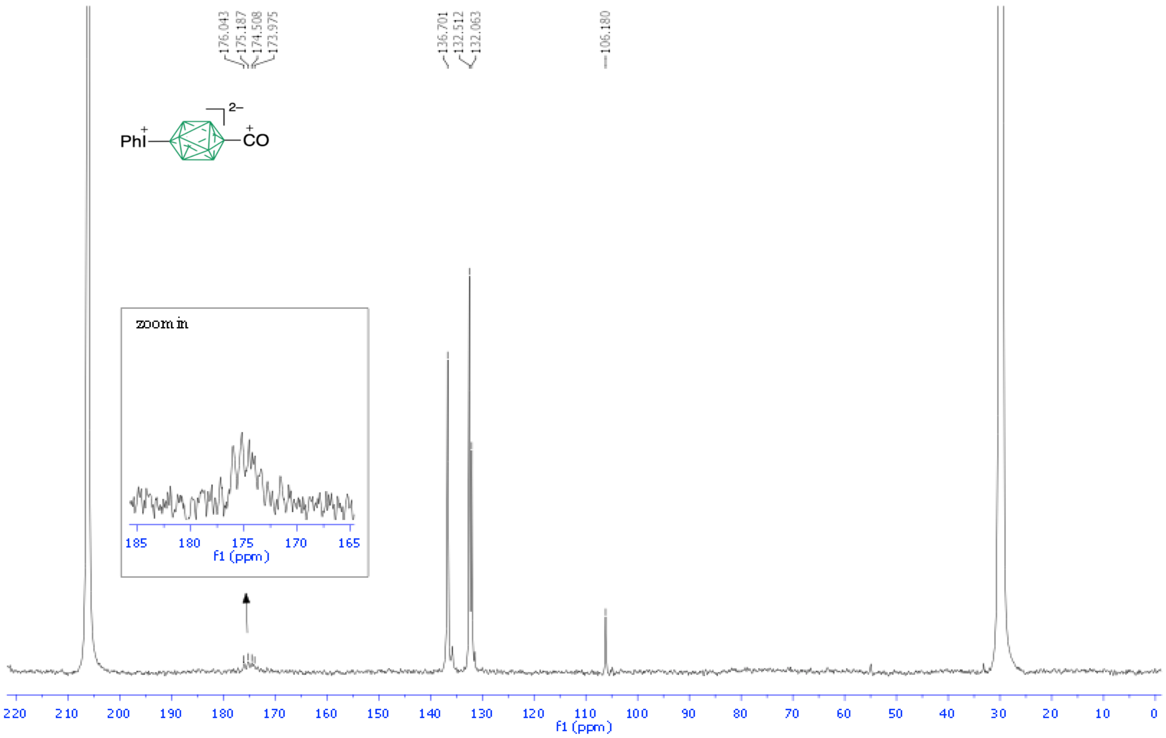

Figure S13. $^{13}C\{^1H\}$ NMR spectrum for crude [*closo*-$B_{10}H_8$-10-IPh-1-CO] (**4**) recorded in acetone-d_6 at 126 MHz.

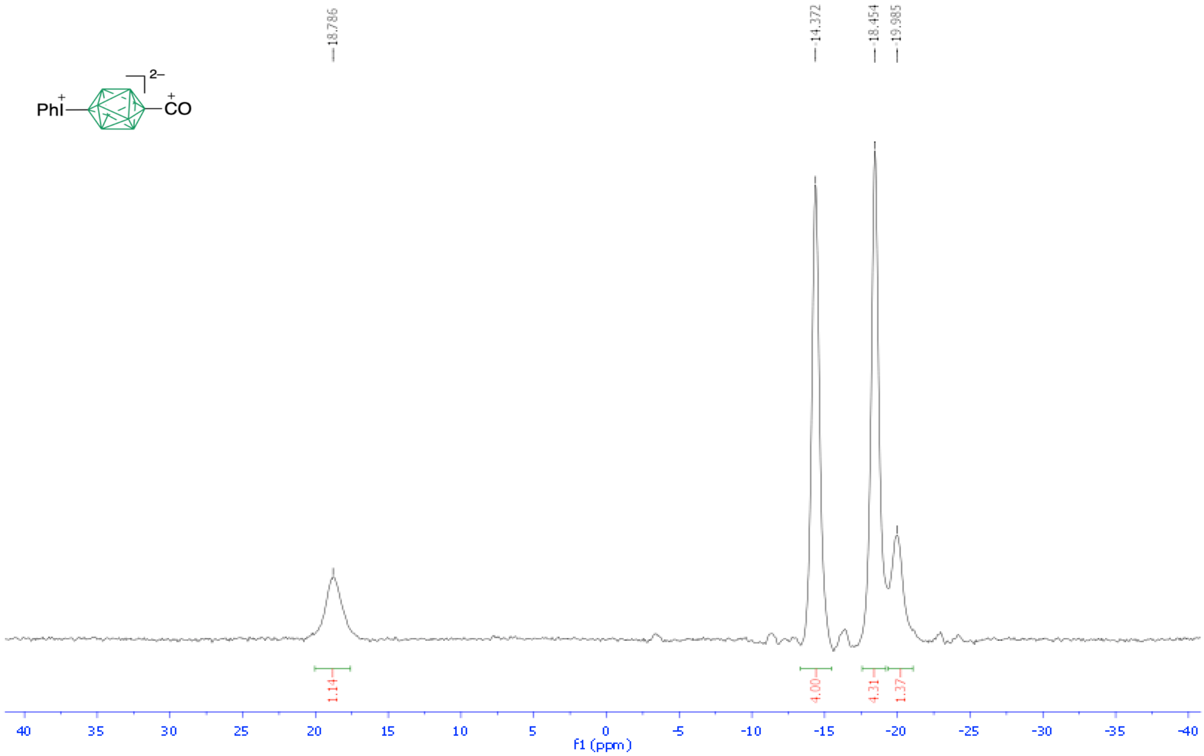

Figure S14. $^{11}B\{^1H\}$ NMR spectrum for crude [*closo*-$B_{10}H_8$-10-IPh-1-CO] (**4**) recorded in acetone-d_6 at 160 MHz.

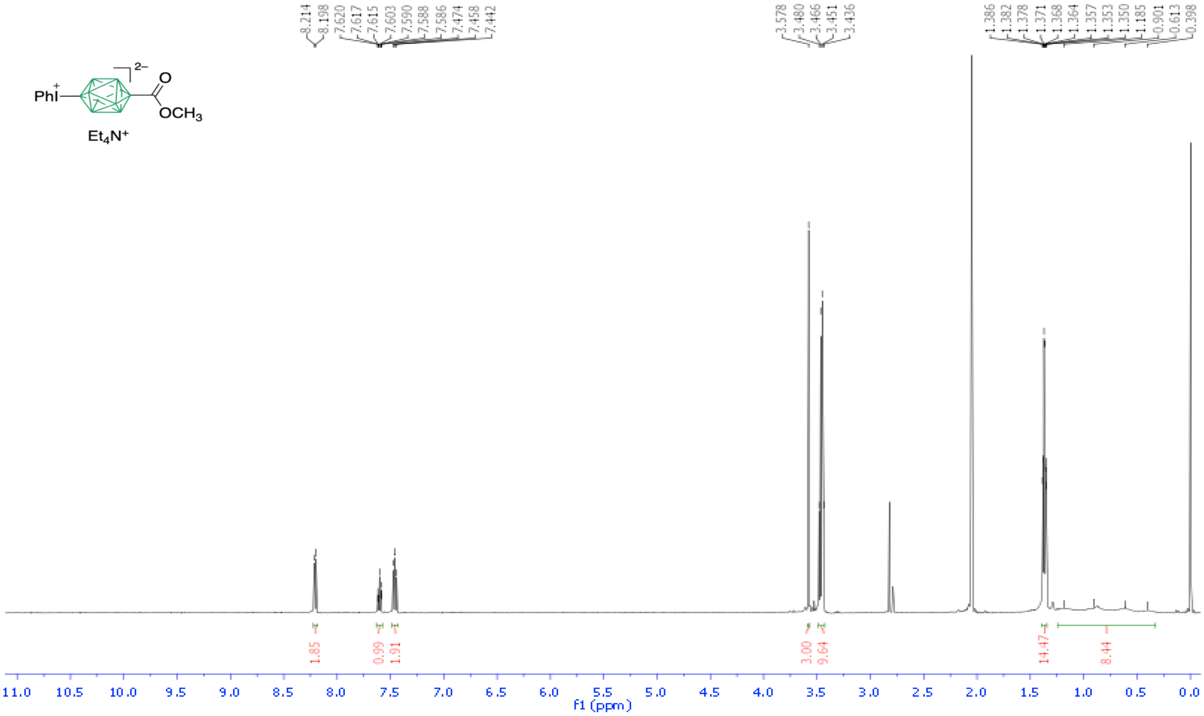

Figure S15. ^{1}H NMR spectrum for [*closo*-$B_{10}H_8$-10-IPh-1-COOMe]$^-$[Et_4N]$^+$ (**5[Et$_4$N]**) recorded in acetone-d_6 at 500 MHz.

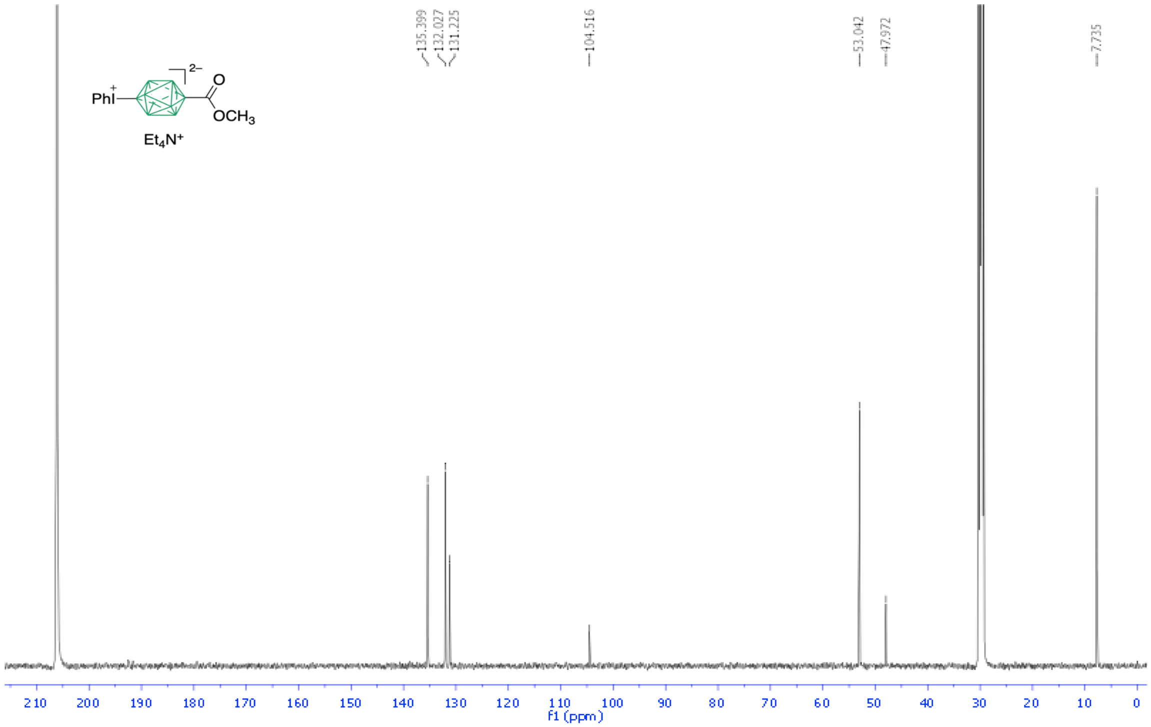

Figure S16. ^{13}C{^{1}H} NMR spectrum for [*closo*-$B_{10}H_8$-10-IPh-1-COOMe]$^-$[Et_4N]$^+$ (**5[Et$_4$N]**) recorded in acetone-d_6 at 126 MHz.

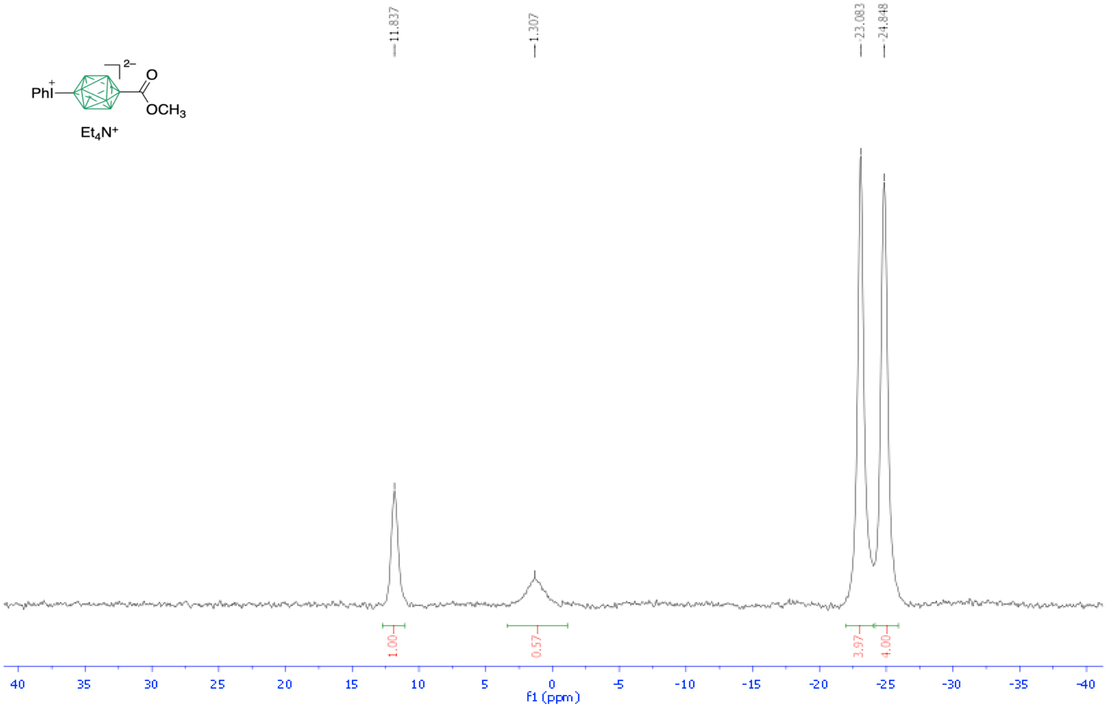

Figure S17. $^{11}B\{^{1}H\}$ NMR spectrum for [*closo*-$B_{10}H_8$-10-IPh-1-COOMe]$^{-}$[Et_4N]$^{+}$ (**5[Et$_4$N]**) recorded in acetone-d_6 at 160 MHz.

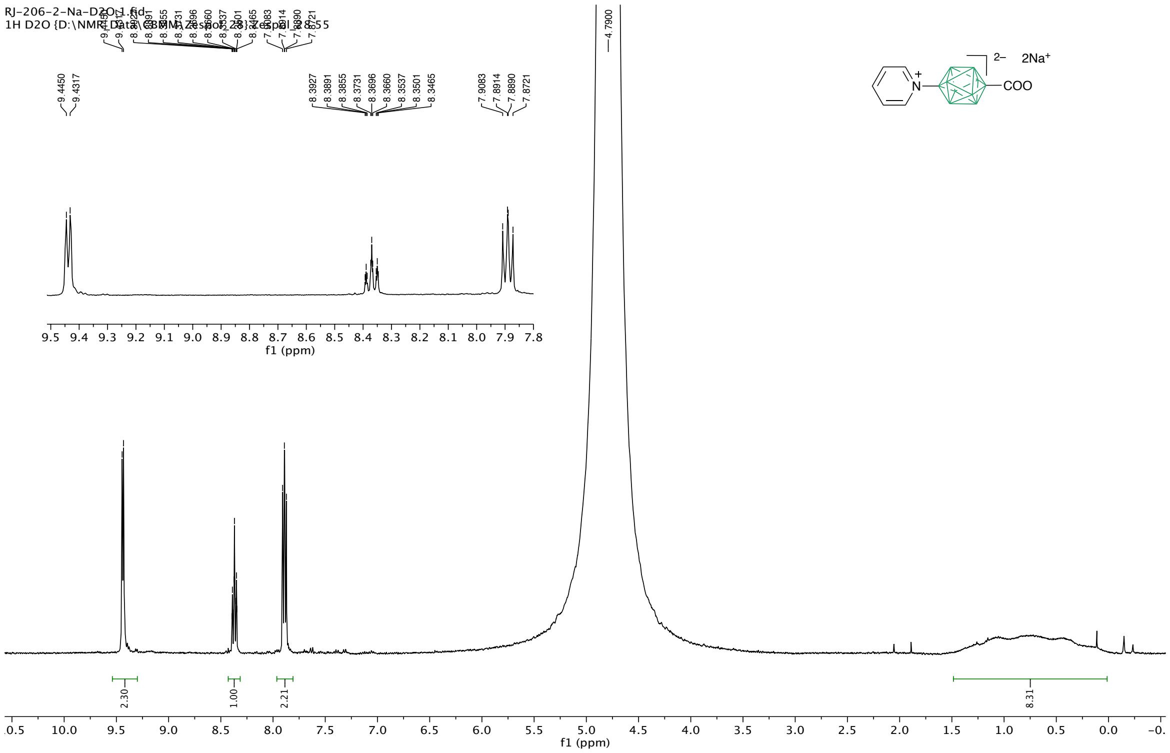

Figure S18. ^{1}H NMR spectrum for crude [*closo*-$B_{10}H_8$-10-C_5H_5N-1-COONa]$^{-}$Na^{+} (**6[2Na]**) recorded in D_2O at 400 MHz.

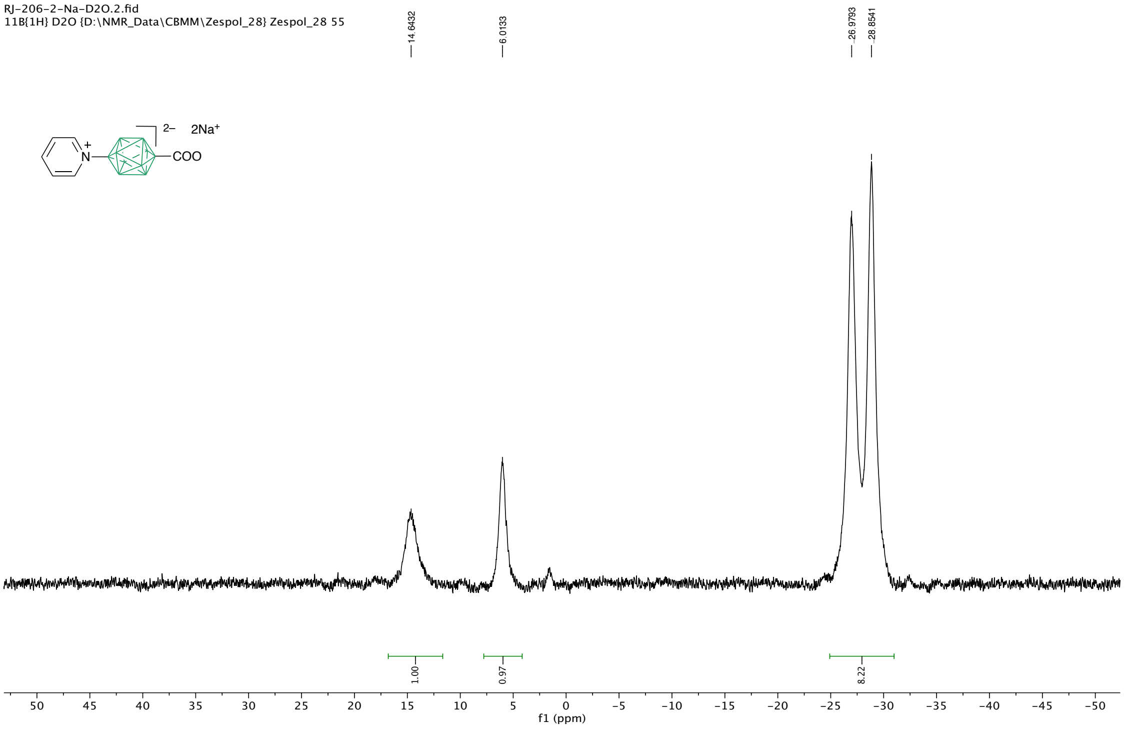

Figure S19. $^{11}B\{^1H\}$ NMR spectrum for crude [*closo*-$B_{10}H_8$-10-C_5H_5N-1-COONa]$^-Na^+$ (**6[2Na]**) recorded in D_2O at 128 MHz.

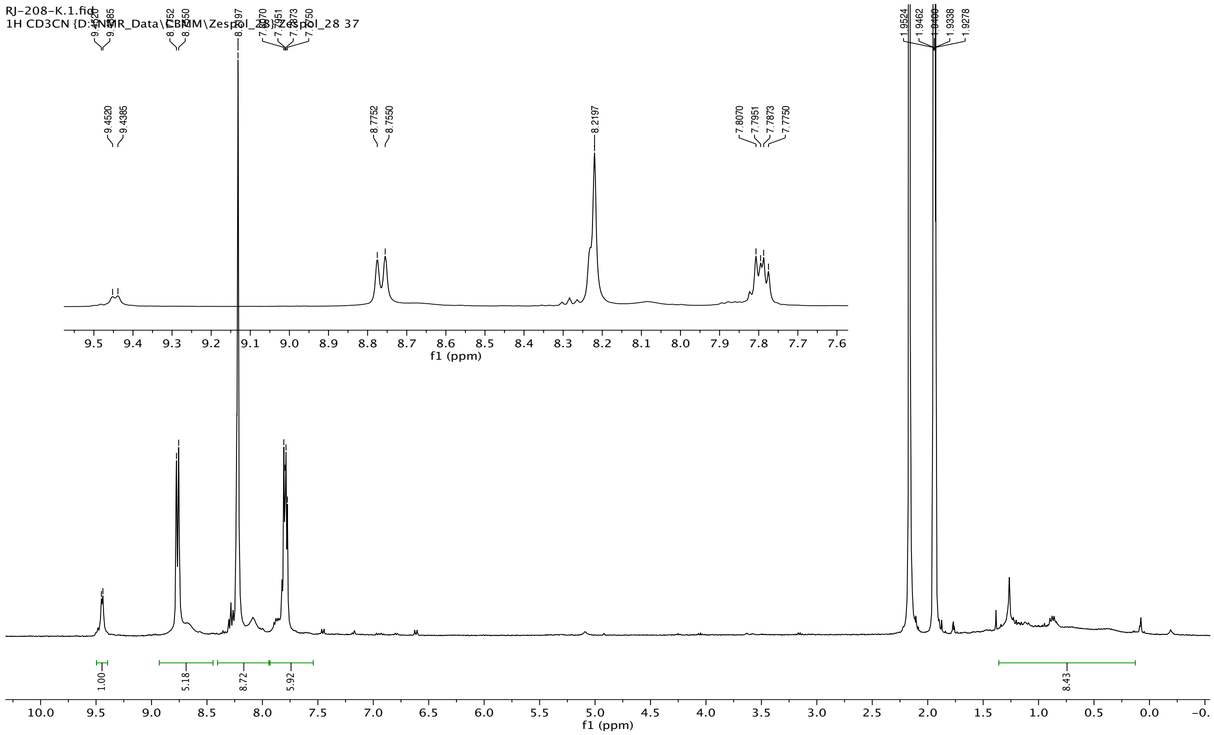

Figure S20. ^1H NMR spectrum for [10-C_5H_5N-*closo*-$B_{10}H_8$-1-COOZn(phen)$_2$] (**6[Zn]**) recorded in CD_3CN at 400 MHz.

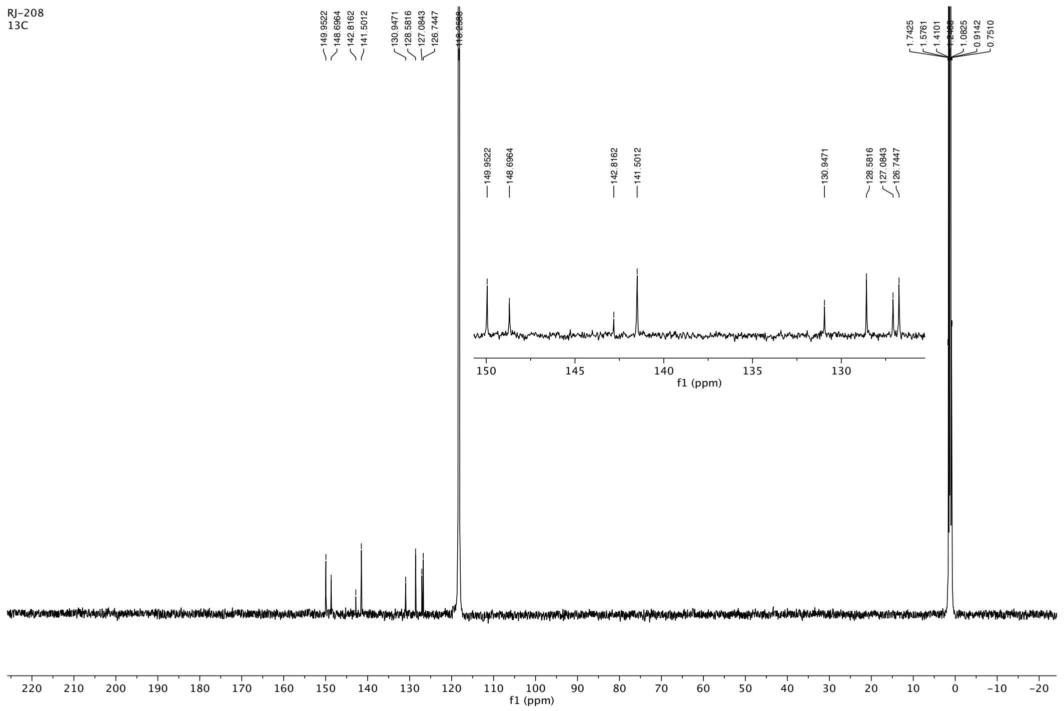

Figure S21. $^{13}C\{^1H\}$ NMR spectrum for [10-C_5H_5N-*closo*-$B_{10}H_8$-1-COOZn(phen)$_2$] (**6[Zn]**) recorded in CD_3CN at 126 MHz.

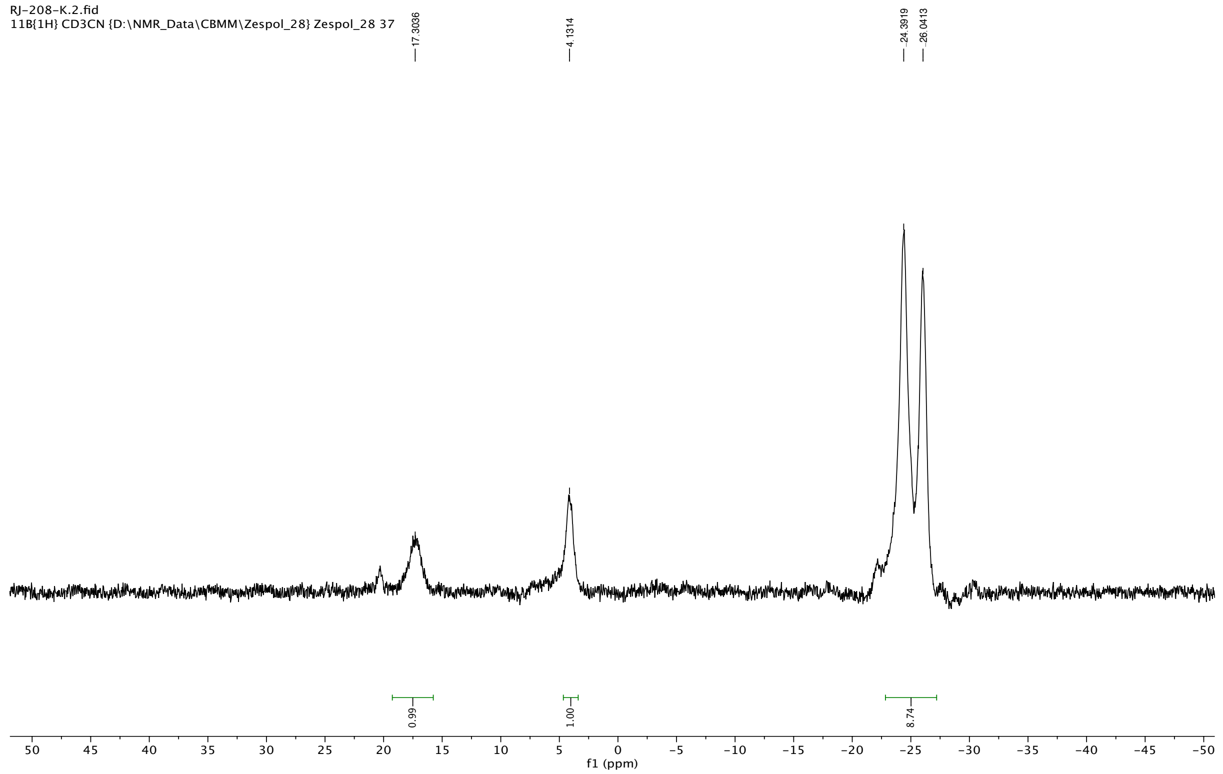

Figure S22. $^{11}B\{^1H\}$ NMR spectrum for [10-C_5H_5N-*closo*-$B_{10}H_8$-1-COOZn(phen)$_2$] (**6[Zn]**) recorded in CD_3CN at 128 MHz.

3. IR spectra

IR spectra for **1f[Et₄N], 6[Zn], 6[Cu],** $Zn(phen)_2(NO_3)_2 \cdot 2H_2O$ and $Cu(phen)_2(NO_3)_2 \cdot H_2O$ were recorded with an ATR attachment for neat solids.

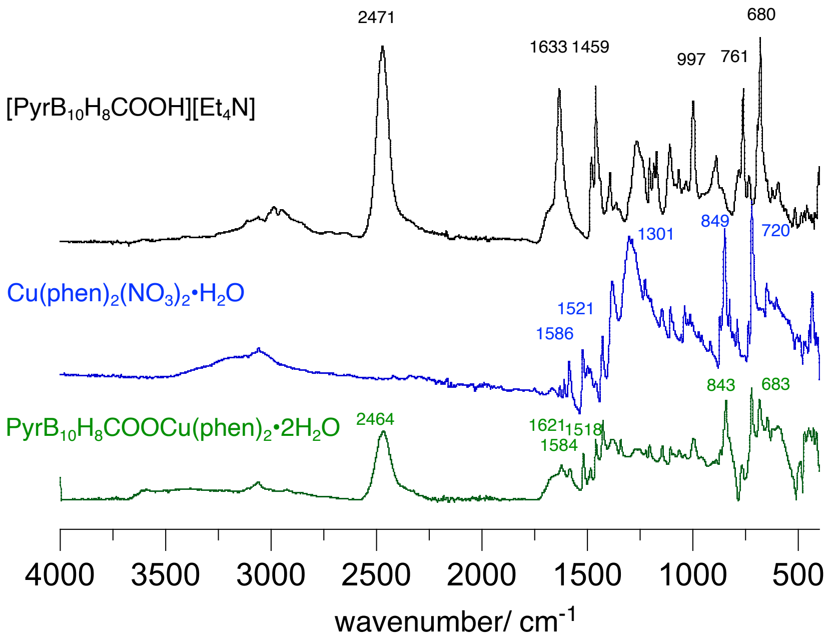

Figure S23. IR spectrum of acid **1f[Et₄N]** (black), $Cu(phen)_2(NO_3)_2 \cdot H_2O$ (blue), and [10-C_5H_5N-*closo*-$B_{10}H_8$-1-COOCu(phen)$_2$] (**6[Cu]**, green) recorded in the solid state.

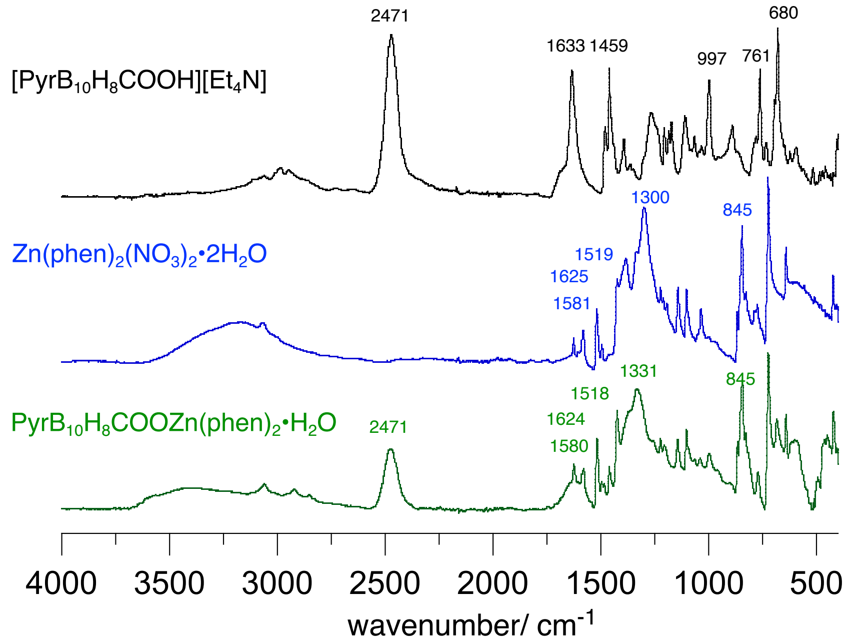

Figure S24. IR spectrum of acid **1f[Et₄N]** (black), $Zn(phen)_2(NO_3)_2 \cdot 2H_2O$ (blue), and [10-C_5H_5N-*closo*-$B_{10}H_8$-1-COOZn(phen)$_2$] (**6[Zn]**, green) recorded in the solid state.

4. UV-vis absorption spectroscopy

For solid-state electronic absorption spectra, MeCN solution of about 1.0 mg of **1f[Et$_4$N]**, **6[Zn]** and Zn(phen)$_2$(NO$_3$)$_2$·2H$_2$O were drop cast onto a quartz plate placed on a hot stage at 80 ^{o}C. After the deposition was finished, the solid material was covered with a second quartz plate, pressed, cooled and placed in a UV spectrometer. Absorbance was measured between 200 and 1000 nm.

Solution absorption spectra of **6[2Na]** and **1f[H$_3$O]** were recorded in a mixture of Milli-Q H$_2$O and spectrophotometric grade MeCN (1:1, *v/v*) using concentration of about 10^{-4} M and then normalized. Absorbance was measured between 200 to 800 nm.

The resulting spectra are shown in Figure 3 in the main text and listed in Section 1 (Synthetic details).

5. XRD data collection and refinement details

Crystal data for **1e[Et$_4$N]** and **3** and were collected on a Rigaku XtaLAB SuperNova, Pilatus 200 K diffractometer at 100 K using micro-focus X-ray Source CuK_α radiation (λ = 1.54184 Å). The data were integrated using CrysAlisPro program.[6] Intensities for absorption were corrected using multi-scan method as in SCALE3 ABSPACK scaling algorithm implemented in CrysAlisPro program.[6] Additional crystal and refinement information are listed in Table S1.

CCDC 2072611 and 2072613 contain the supplementary crystallographic data for this paper. These data can be obtained free of charge from The Cambridge Crystallographic Data Centre via www.ccdc.cam.ac.uk/structures.

Structure solution and refinement

The structures were solved with the ShelXT structure solution program[7] using Intrinsic Phasing and refined in the ShelXL by the full-matrix least-squares minimization on F^2 with the ShelXL refinement package.[8] All non-hydrogen atoms were refined anisotropically and C–H hydrogens were generated geometrically using the HFIX command as in ShelXL. Hydrogen atoms were refined isotropically and constrained to ride on their parent atoms. The crystal data and structure refinement descriptors are presented in Table S1. Partial packing diagrams are shown in Figures S25–S30.

Table S1. Selected structural data for compounds 1e[Et_4N] and 3.

	**1e[Et_4N]**<br>CCDC: 2072613	**3**<br>CCDC: 2072611
Formula	$C_{15}H_{34}B_{10}INO_2$	$C_{8.4}H_{16.8}B_{10}Cl_{0.8}IN$
Formula Weight	495.43	395.19
Crystal System	Orthorhombic	Monoclinic
Space Group	*Pbca*	*C*2/*c*
a/Å	17.6587(3)	32.2967(6)
b/Å	15.1042(3)	8.84864(16)
c/Å	18.4369(3)	12.1104(2)
α/°	90	90
β/°	90	99.8950(17)
γ/°	90	90
Volume/Å^3	4917.50(15)	3409.44(11)
Z	8	8
2θ range for data collection/°	4.538 to 76.703	2.778 to 76.374
Index ranges	$-17 \leq h \leq 21$, $-17 \leq k \leq 18$, $-23 \leq l \leq 19$	$-40 \leq h \leq 39$, $-10 \leq k \leq 5$, $-15 \leq l \leq 13$
No. of measured, independent, and observed [$I>2\sigma(I)$] reflections	22512, 5091, 4767	8031, 3436, 2961
*R*int	0.0485	0.0354
Goodness-of-fit on F^2	1.032	1.054
Final *R* indexes [$F^2 > 2\sigma(F^2)$]	R_1=0.0420, wR_2=0.1193	R_1=0.0504, wR_2=0.1406
Final *R* indexes [all data]	R_1=0.0436, wR_2= 0.1209	R_1=0.0567, wR_2= 0.1454
Data/restraints/parameters	5091/0/267	3436/1/205
Largest diff. peak/hole Å^{-3}	1.391/-1.185	2.223/-1.343

Table S2. Selected interatomic distances and angles for derivatives **A[Q]**, **1e[Et$_4$N]** and **3**.[a]

	**A[Q]** [b]	**1e[Et$_4$N]**	**3**
X =	H	COOH	CNMe
X–B(1)	–	1.582(4)	1.541(11)
B(1)–B(2)$_{avrg}$	1.701(3)	1.696(1)	1.684(13)
B(1)···B(2-5) [c]	1.100	1.084	1.049
B(2)–B(3) $_{avrg}$	1.835(9)	1.845(10)	1.864(11)
B(2)–B(6) $_{avrg}$	1.813(6)	1.810(8)	1.810(12)
B(6)–B(7)$_{avrg}$	1.835(9)	1.865(9)	1.861(12)
B(10)···B(6-9) [c]	1.100	1.039	1.021
B(10)–B(9)	1.701(3)	1.678(5)	1.665(10)
PhI–B(10)	–	2.179(3)	2.190(7)
B–B(1)–X $_{avrg}$	130.3(12)	129.7(8)	128(2)
B–B(10)–I $_{avrg}$	130.3(12)	128(3)	127.8(5)
B(10)–I–C	–	101.1(1)	95.9(2)
B(1)···B(10)	3.717(4)	3.629(4)	3.57(1)

[a] All distances are in Å and angles in degrees. Except for unique in each molecule distances B−X and the cage size B(1)···B(10), all parameters are average values and the esd refers to the distribution of individual values. [b] Q = 2,2′-bipyridinium, ref. [9] [c] The height of the square pyramid.

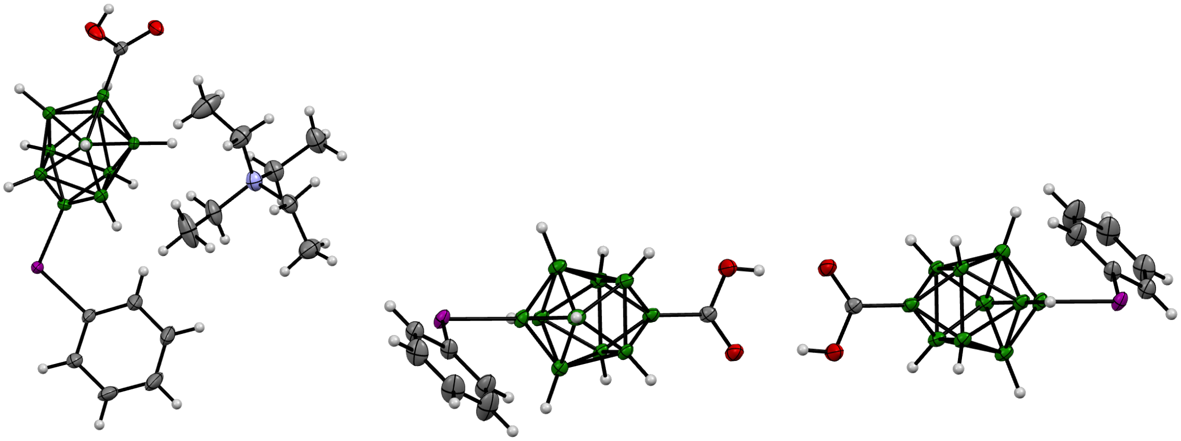

Figure S25. Atomic displacement ellipsoid representations of ion pair **1e[Et$_4$N]** (left) and carboxylic acid dimer with the O···O separation of 2.694(1) Å (right). Thermal ellipsoids are at the 50% probability level. For geometrical dimensions see Table S2.

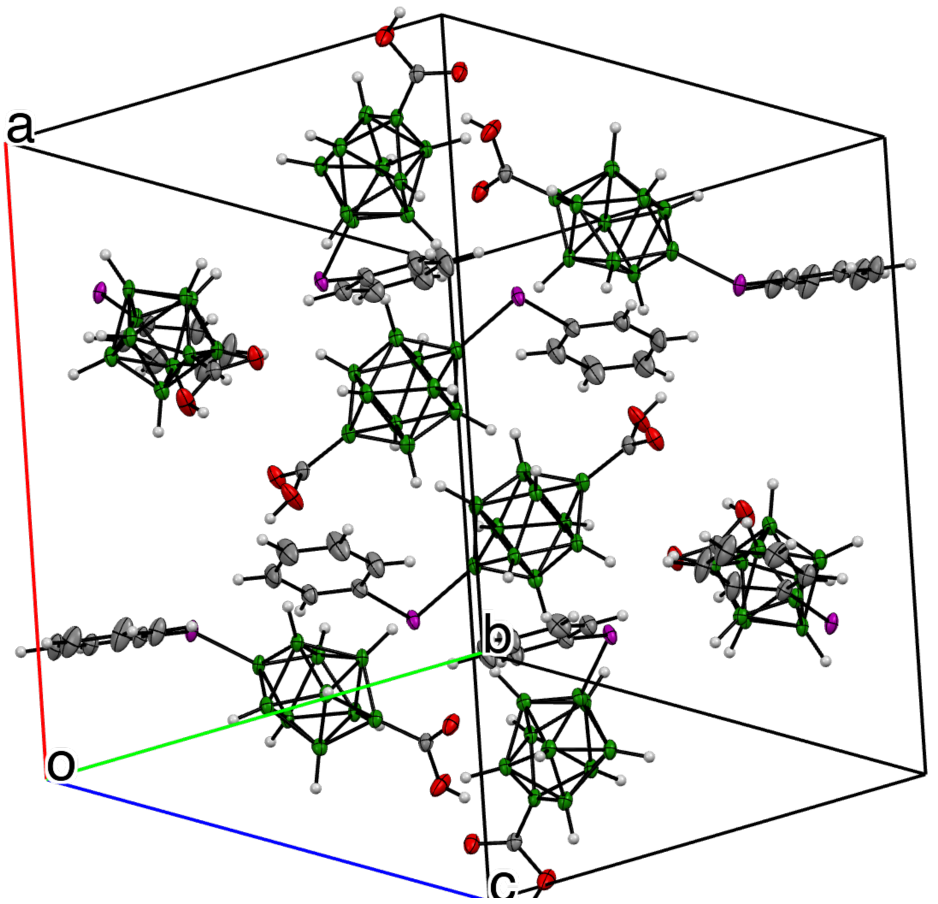

Figure S26. Unit cell packing diagram for **1e[Et_4N]**. The $[Bu_4N]^+$ cations are omitted for clarity. Thermal ellipsoids are at the 50% probability level.

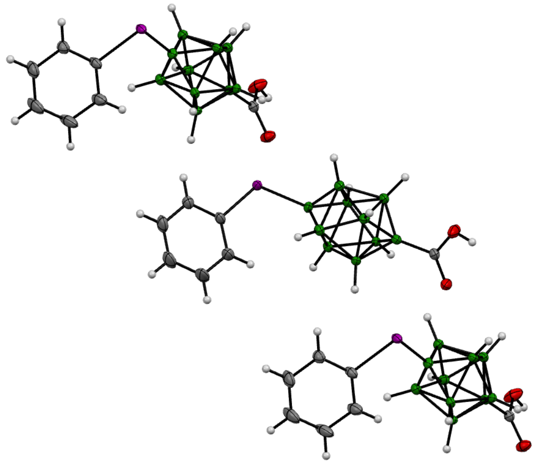

Figure S27. Part of infinite chain of close nonbonding B···I interactions 3.625 and 3.675 Å, which are 0.355 and 0.305 Å inside the van der Waals separation. Thermal ellipsoids are at the 50% probability level.

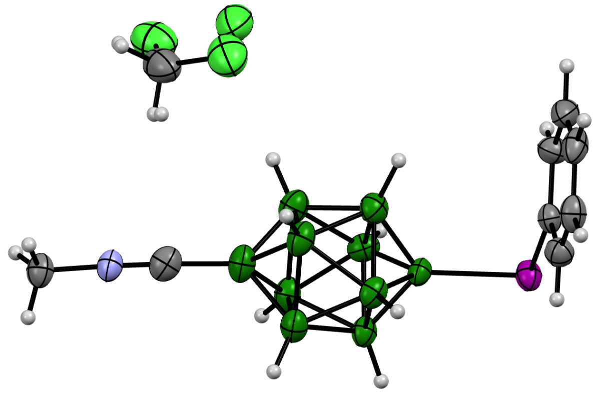

Figure S28. Atomic displacement ellipsoid representations **3** and disordered molecule of CH_2Cl_2. Thermal ellipsoids are at the 50% probability level. For geometrical dimensions see Table S2.

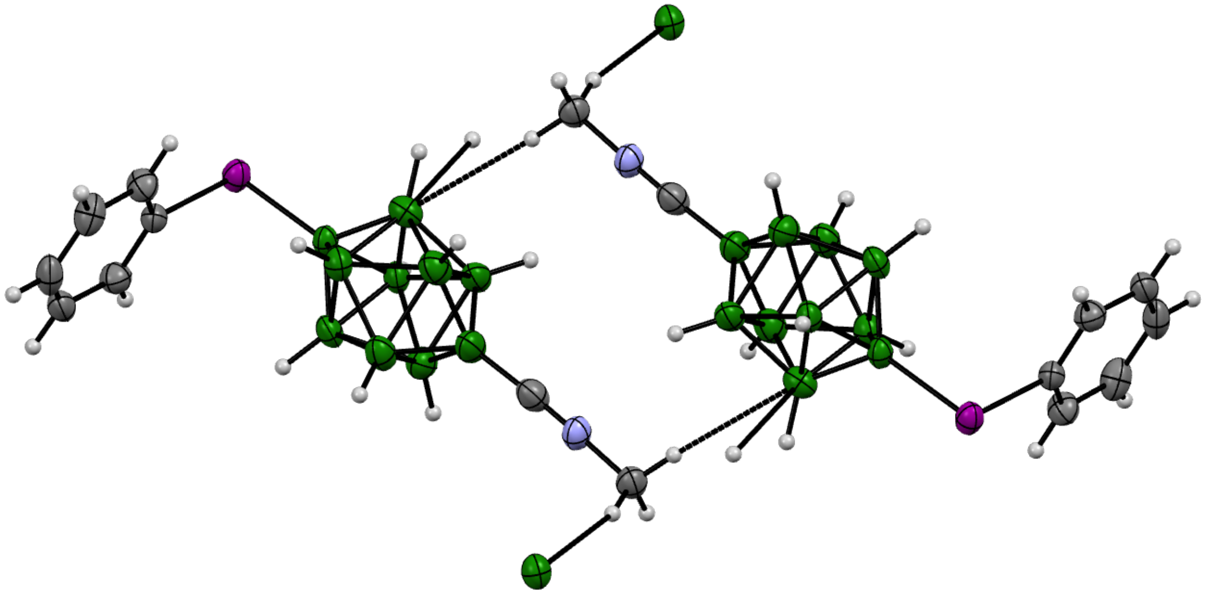

Figure S29. A discrete dimer of **3** with close B···HC nonbonding distance. Thermal ellipsoids are at the 50% probability level.

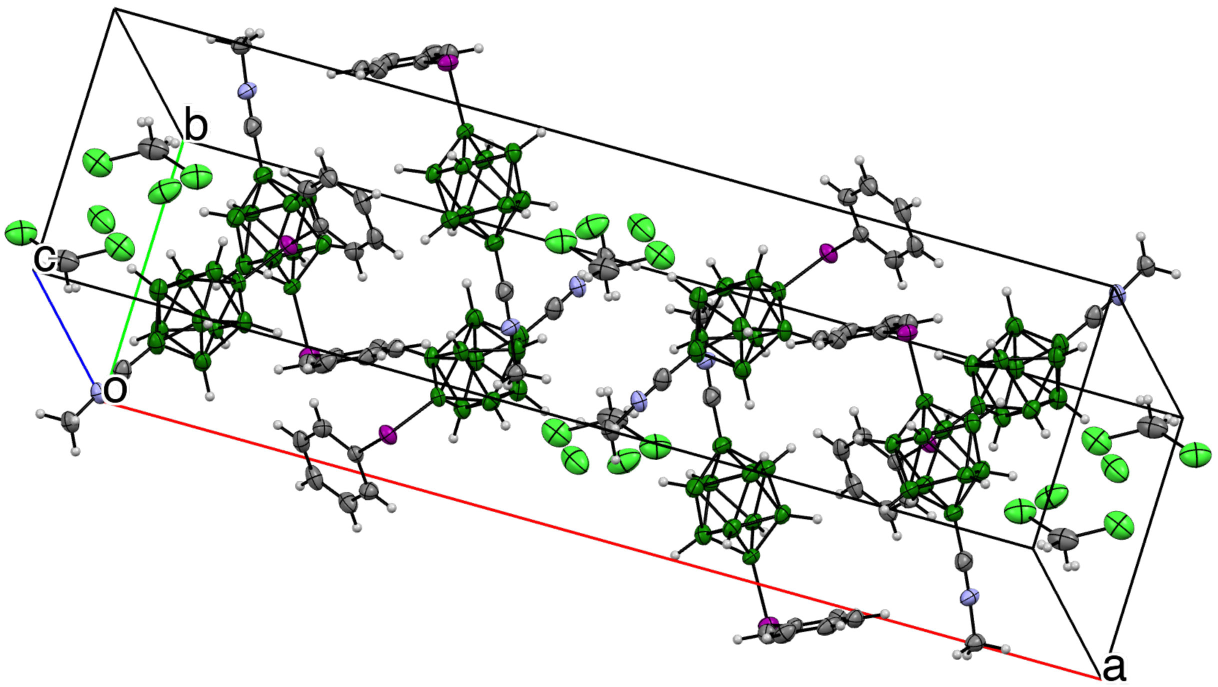

Figure S30. Unit cell packing diagram for **3** with positionally disordered CH_2Cl_2 molecules.

6. Attempts at growing crystals of metal complexes 6[M]

Samples of **6[Cu]** were attempted to recrystallize from the following solvent systems: MeCN, MeCN/H_2O, acetone/H_2O/MeCN, DCM/MeCN (layered), EtOH, MeOH, MeOH/MeCN/H_2O, DMA/H_2O or MeCN/H_2O (slow crystallization using a Dewar vessel with hot water). Crystalline-like material of **6[Cu]** was obtained only using hot MeCN with a small amount of water (Figure S31), however the sample was also moderately soluble in other solvents on heating, excluding alcohols.

Similar methods were tested for recrystallization of **6[Zn]**. Due to its poor solubility in most solvents, only hot MeCN with a small amount of water was found to give the powdery material after recrystallization (Figure S31).

Additionally, hydrothermal recrystallization was also tested. Thus, 5 or 10 mg sample of **1f[Et$_4$N], 6[2Na]** or **1f[H$_3$O]** were placed in a pressure Teflon vessel together with $Zn(phen)_2(NO_3)_2{\cdot}2H_2O$ or $Cu(phen)_2(NO_3)_2{\cdot}H_2O$ (1 equiv.) and 2 mL of H_2O or mixtures of MeCN and H_2O (different ratios). The vessel was sealed, mounted in the oven and heated for 24 h at 100 °C and then cooled down at a rate of 5 K/1 h. Mixing **1f[Et$_4$N], 6[2Na]** or **1f[H$_3$O]** with different copper(II) salts (i.e. $CuCl_2{\bullet}2H_2O$, $Cu(OAc)_2$) and ligands, such as 1,10-phenantroline,

TMEDA or 2,2'-bipyridine in 2 mL of H_2O or mixtures of MeCN and H_2O (different ratios) for the hydrothermal recrystallization was also investigated. All attempts to grow a single crystal of **6[Cu]** or **6[Zn]** were unsuccessful to produce XRD quality crystals.

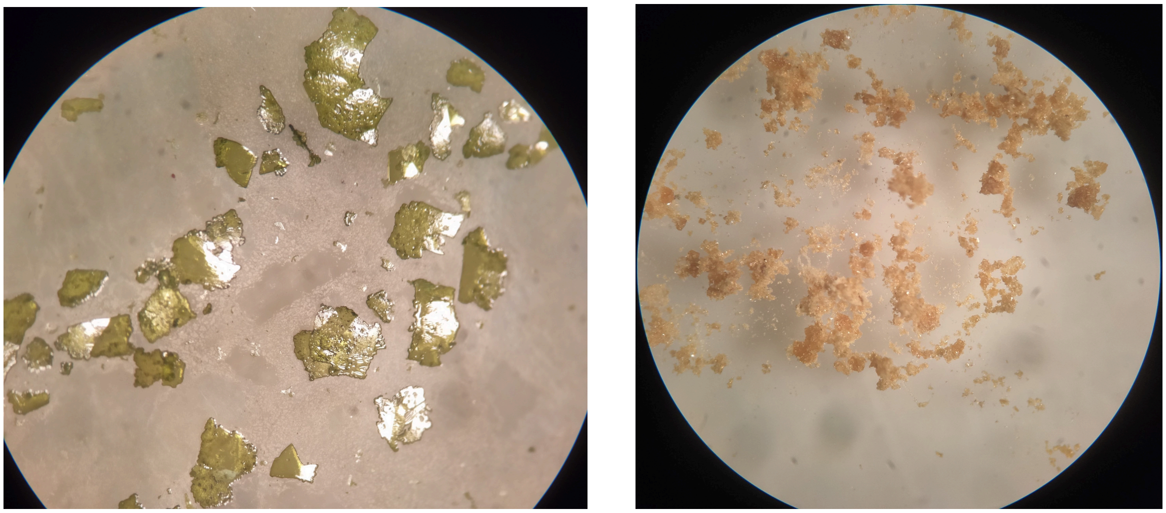

Figure S31. Typical solid material obtained in an attempted crystallization of **6[Cu]** (left) and **6[Zn]** (right).

7. References

(1) Hawthorne, M. F.; Pilling, R. L.; Knoth, W. H. Bis(triethylammonium) decahydrodecaborate(2-), *Inorg. Synth.* **1967**, *9*, 16–19.

(2) Fulmer, G. R.; Miller, A. J. M.; Sherden, N. H.; Gottlieb, H. E.; Nudelman, A.; Stoltz, B. M.; Bercaw, J. E.; Goldberg, K. I. NMR chemical shifts of trace impurities: Common laboratory solvents, organics, and gases in deuterated solvents relevant to the organometallic chemist, *Organometallics* **2010**, *29*, 2176–2179.

(3) Kapuściński, S.; Abdulmojeed, M. B.; Schafer, T. E.; Pietrzak, A.; Hietsoi, O.; Friedli, A. C.; Kaszyński, P. Photonic materials derived from the [*closo*-$B_{10}H_{10}]^{2-}$ anion: Tuning photophysical properties in [*closo*-$B_{10}H_8$-1-X-10-(4-Y-NC_5H_5)$]^-$, *Inorg. Chem. Front.* **2021**, *8*, 1066–1082.

(4) Nakai, H.; Deguchi, Y. The crystal structure of monoaquobis(1,10-phenanthroline)copper(II) nitrate, $[Cu(H_2O)(phen)_2](NO_3)_2$, *Bull. Chem. Soc. Jpn.* **1975**, *48*, 2557–2560.

(5) Lai, J. W.; Maah, M. J.; Tan, K. W.; Sarip, R.; Lim, Y. A. L.; Ganguly, R.; Khaw, L. T.; Ng, C. H. Dinuclear and mononuclear metal(II) polypyridyl complexes against drug-sensitive and drug-resistant Plasmodium falciparum and their mode of action, *Malar. J.* **2022**, *21*, 386.
(6) CrysAlisPro 1.171.40.84a, Rigaku Oxford Diffraction (2020).
(7) Sheldrick, G. M. SHELXT – Integrated space-group and crystal- structure determination, *Acta Crystallogr., Sect. A* **2015**, *A71*, 3-8.
(8) Sheldrick, G. M. SHELXT – Integrated space-group and crystal- structure determination, *Acta Crystallogr., Sect. C* **2015**, *C71*, 3-8.
(9) Mebs, S.; Kalinowski, R.; Grabowsky, S.; Förster, D.; Kickbusch, R.; Justus, E.; Morgenroth, W.; Paulmann, C.; Luger, P.; Gabel, D.; Lentz, D. Real-space indicators for chemical bonding. Experimental and theoretical electron density studies of four deltahedral boranes., *Inorg. Chem.* **2011**, *50*, 90–103.
